# Impact of Psychological Stress and Spontaneous Tumour Regression on the Hippocampal Proteome in a Mouse Model of Breast Cancer

**DOI:** 10.1111/jnc.70052

**Published:** 2025-04-02

**Authors:** Myrthe Mampay, Gheed Al‐Hity, Sara O. Rolle, Walla Alzboon, Nicolas A. Stewart, Melanie S. Flint, Graham K. Sheridan

**Affiliations:** ^1^ School of Applied Sciences University of Brighton Brighton UK; ^2^ Green Templeton College University of Oxford Oxford UK; ^3^ School of Life Sciences University of Nottingham Nottingham UK

**Keywords:** breast cancer, cytokines, hippocampus, proteomics, stress, tumour regression

## Abstract

Cognitive impairment is common in people diagnosed with breast cancer, but the molecular mechanisms that underlie maladaptive changes in the brain are unknown. The psychological stress of a cancer diagnosis is certainly a contributing factor. Here, we investigated alterations in the hippocampal proteome in response to both cancer and psychological stress using label‐free quantitative mass spectrometry techniques. An orthotopic syngeneic model of triple‐negative breast cancer (TNBC) was established by injecting Py230 cells into the mammary fat pads of female C57Bl/6 mice. Half of the mice were subjected to a daily restraint stress paradigm. Mice that experienced both cancer and restraint stress lost weight and displayed larger tumours compared to non‐stressed mice. Their urinary corticosterone levels were also elevated, as measured by enzyme‐linked immunosorbent assay. Non‐stressed tumour‐bearing mice displayed higher levels of TNFα in the prefrontal cortex (PFC) compared to stressed mice with cancer. Flow cytometry results suggested that the CD4^+^/CD8^+^ T cell ratios were also raised in non‐stressed tumour‐bearing mice compared to both controls and stressed mice with TNBC. Bioinformatic analysis of hippocampal proteomes indicated that cancer alone causes reduced mitochondrial respiration and ATP synthesis, as well as impaired glutamate recycling and synaptic plasticity. Moreover, daily stress in TNBC mice caused further mitochondrial dysfunction, increased oxidative phosphorylation, and altered lipid metabolism. Importantly, over half of the mammary tumours that initially developed spontaneously regressed after 7–9 weeks in these young immunocompetent mice. Tumour regression inhibited TNFα increases in the PFC. However, the hippocampal proteomes of tumour‐bearing mice were largely similar to mice in which tumours regressed, suggesting that spontaneous regression of breast cancer confers lasting physiological dysregulations that impact hippocampal protein expression. This study in mice may help to identify molecular mechanisms responsible for long‐term memory impairments in cancer survivors and reveal novel drug targets for cancer‐related cognitive impairment.

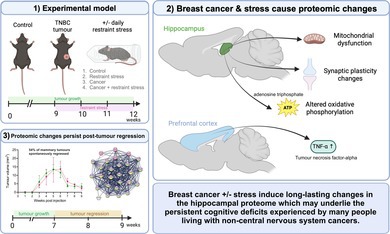

## Graphical abstract

This study examines how breast cancer and psychological stress impact the hippocampal proteome, uncovering altered signalling pathways linked to mitochondrial dysfunction, impaired energy production, and altered synaptic plasticity; that is, cellular processes that are critical for memory formation and cognitive health. Using a triple‐negative breast cancer (TNBC) mouse model paired with chronic restraint stress, our findings also suggest that non‐CNS tumour regression causes enduring molecular changes in the hippocampus. By identifying these protein‐level disruptions, this work advances our understanding of cancer‐related cognitive impairment, distinct from treatment effects, and highlights the long‐lasting neurological impact of cancer itself on brain health.

**Abbreviations**
7‐AAD: 7‐amino‐actinomycin D
ADP: adenosine di‐phosphate
AGC: automatic gain control
Amb: ammonium Bicarbonate
AMPA: α‐amino‐3‐hydroxy‐5‐methyl‐4‐isoxazolepropionic acid
ATP: adenosine tri‐phosphate
AU: Airy Unit
BBB: blood brain barrier
BDNF: brain‐derived neurotrophic factor
BP: biological process
BSA: bovine serum albumin
cAMP: cyclic adenosine monophosphate
CAN: cancer
CC: cellular components
CNS: central nervous system
CRCI: cancer‐related cognitive impairment
CREB: cAMP‐response element binding protein
CRS: cancer + restraint stress
CTL: cytotoxic T lymphocytes
DAPI: 4′,6‐diamidino‐2‐phenylindole
DEP: differentially expressed protein
DMSO: dimethyl sulfoxide
DTT: 1,4‐Dithiothreitol
EAAT: excitatory amino acid transporters
EDTA: ethylenediaminetetraacetic acid
ELISA: enzyme‐linked immunosorbent assay
FACS: fluorescence activated cell sorting
FBS: foetal bovine serum
FDR: false discovery rate
FGF: fibroblast growth factor
GABA: gamma‐aminobutyric acid
GFAP: glial fibrillary acidic protein
GO: gene ontology
GTP: guanosine triphosphate
HCD: higher‐energy collisional dissociation
HPA: hypothalamic–pituitary‐adrena
IAA: iodoacetamide
ICAM‐1: intercellular adhesion molecule 1
ICD: immunogenic cell death
IFN‐γ: interferon‐γ
IP3R: inositol triphosphate receptor
KEGG: Kyoto Encyclopaedia of Genes and Genomes
LFQ: label‐free quantification
LOD: limit of detection
LTP: long‐term potentiation
MAM: mitochondria‐associated ER membrane
MAPK: mitogen‐activated protein kinases
MF: molecular functions
MHC: major histocompatibility complex
MMTV‐PyMT: mammary‐specific polyomavirus middle T antigen overexpression mouse model
MS: mass spectrometry
nLC‐MS/MS: nanoflow liquid chromatography mass spectrometry
NMDA: N‐methyl‐D‐aspartate
PBS: phosphate‐buffered saline
PCA: principal component analysis
PFC: prefrontal cortex
PKA: protein kinase A
PPAR: peroxisome proliferator‐activated receptors
qPCR: quantitative polymerase chain reaction
RNA: ribonucleic acid
ROS: reactive oxygen species
RPMI: Roswell Park Memorial Institute
RS: restraint stress
TFA: trifluoroacetic acid
TGF‐β: transforming growth factor‐β
TNBC: triple negative breast cancer
TNFα: tumour necrosis factor alpha
Tregs: regulatory T cells
TTR: transthyretin

## 1 Introduction

Cognitive disturbances are common among people diagnosed with non‐central nervous system (non‐CNS) cancers, as well as those who are in disease remission (Schmidt et al. 2016). The condition, often termed cancer‐related cognitive impairment (CRCI), was initially attributed to chemotherapy‐associated toxicity and is sometimes referred to as ‘*chemobrain*’. However, up to 40% of patients with non‐CNS cancers experience impaired cognitive performance prior to receiving any surgery or anti‐neoplastic treatments (Ahles et al. 2008; Cimprich et al. 2010; Hardy et al. 2018; Janelsins et al. 2014). Therefore, to distinguish the condition *chemobrain* from earlier pre‐treatment cognitive deficits caused by non‐CNS tumour growth, the term ‘*tumour brain*’ has more recently been coined. Pre‐treatment cognitive impairment is typically characterised by deficits in executive functions, attention, information processing speed, and short‐ and long‐term memory retention (Ahles et al. 2008; Berman et al. 2014; Jansen et al. 2011; Vardy et al. 2008). Cognitive dysfunctions in cancer patients, whom already experience significant concerns about their disease and future outlook, can have serious deleterious effects on their emotional wellbeing, ability to work, self‐care, social life, treatment adherence and overall quality of life (Olson and Marks 2019).

Currently, little is known about the aetiology of pre‐treatment CRCI (Koleck et al. 2014; Vardy et al. 2015). However, the existence of cognitive dysfunction prior to the commencement of anti‐neoplastic therapy suggests that tumour‐derived factors play a role, either directly or indirectly, in modulating cognition (Andreotti et al. 2015; Mampay et al. 2021; Olson and Marks 2019). For instance, cancer‐induced dysfunction of the immune system could lead to a state of chronic low‐level neuroinflammation in key cognitive brain regions, such as the hippocampus and prefrontal cortex (Lomeli et al. 2021). Moreover, patients often report feeling significantly higher stress levels after receiving a cancer diagnosis due to the uncertainty about their future and anxiety about having to undergo harsh treatment regimens (Papanastasiou et al. 2019). The physiological response to this kind of psychological stress is in part mediated via activation of the hypothalamic–pituitary–adrenal (HPA) axis, leading to the production of catecholamines (e.g., norepinephrine and epinephrine) and glucocorticoids (e.g., cortisol). If activated chronically, high glucocorticoid levels cause a state of systemic inflammation and promote increased release of proinflammatory cytokines from immune cells (Walsh et al. 2021). Thus, cancer‐related immune dysfunction could synergise with diagnosis‐associated psychological stress to impair cognitive functioning via maladaptive signalling between the immune and central nervous systems (Liu et al. 2022). To date, changes to diurnal cortisol rhythms, blunted cortisol rises in response to stress, and glucocorticoid receptor resistance have all been reported in cancer patients and survivors (Andreotti et al. 2015; Bower et al. 2011). Although these physiological changes have not been directly linked to pre‐treatment CRCI, it is likely that people living with cancer display aberrant responses to stress which exacerbate cognitive impairments (Mitchell et al. 2013). However, the lack of basic mechanistic research on pre‐treatment cognitive dysfunction in non‐CNS cancers makes it difficult to determine the contribution of psychological stress versus pathophysiological factors in the initiation and progression of CRCI.

Here, our aim was to develop an in vivo syngeneic mouse model of non‐metastatic breast cancer which could be used to investigate the impact of non‐CNS tumour growth on both neuroinflammation and on global proteomic changes in the hippocampus; a brain region known for its importance in learning and memory formation (Bird and Burgess 2008). Importantly, we combined this mouse model with and without a well‐characterised daily psychological (restraint) stress paradigm with the aim of mimicking the chronic diagnosis‐associated stress felt by humans living with cancer (Wu et al. 2022). Although not a perfect representation of the human condition, this model allowed us to selectively discriminate between tumour‐ and stress‐induced changes to the hippocampal proteome, as well as their combined impact. From a mechanistic standpoint, this is advantageous since it is often not possible to investigate the pathophysiological factors that lead to cognitive impairment in patient cohorts in the absence of significant confounding factors, such as diagnosis‐associated distress or anti‐neoplastic treatment regimens. Moreover, a key driving force for this study is to guide future drug discovery efforts aimed at identifying novel molecular targets for minimising CRCI and enhancing the quality of life of cancer survivors.

## 2 Materials and Methods

### 2.1 In Vivo Breast Cancer Model

#### 2.1.1 Ethics Statement

This study was not pre‐registered. All experiments involving animals and schedule 1 protocols were approved by the Animal Welfare and Ethical Review Body (AWERB committee) of the University of Brighton and carried out under UK Home Office licence‐approved protocols (PiL: 70/8361; PpL: 14CAFBE76). This study was conducted in accordance with the principles of the Basel Declaration and adhered to the legislation detailed in the UK Animals (Scientific Procedures) Act 1986 amendment regulations (SI 2012/3039). All efforts were taken to maximise animal welfare conditions and reduce the number of animals used in accordance with the European Communities Council Directive of 20th September 2010 (2010/63/EU).

#### 2.1.2 Syngeneic Triple‐Negative Breast Cancer Model

Six week old female C57Bl/6J mice (Charles River, UK) were aged for 6 weeks in standard open‐top laboratory cages containing a red‐tinted polycarbonate shelter and bedding material until they were 3 months of age (12 weeks), prior to the commencement of any procedures. They were group housed with three to five mice per cage and the environmental conditions of the room were maintained at 19°C ± 1°C and 55% humidity. Mice were fed on a standard maintenance diet and given *ad libitum* access to food and water. For 2 weeks prior to all experimental tasks, animals were handled daily by the same researcher and habituated to the experimental room. The triple‐negative breast cancer (TNBC) mouse model was generated using the C57Bl/6 MMTV‐PyMT syngeneic cell line, Py230, purchased from ATCC (RRID: CVCL_AQ08). This cell line is not listed as a commonly misidentified cell line according to the International Cell Line Authentication Committee (ICLAC). As the distributor of the Py230 cell line, ATCC performs routine authentication using short tandem repeat (STR) profiling. No further authentication was conducted after purchase. However, cell cultures were continually monitored for morphology, growth characteristics, and mycoplasma contamination. Cells were cultured in Ham's F‐12K (Kaighn's) culture medium (Thermo Fisher, UK) supplemented with 5% foetal bovine serum (FBS) (Thermo Fisher, UK) and 0.1% MITO+ Serum Extender (Corning, Thermo Fisher, UK) at 37°C and 5% CO_2_. At 3 months of age, half of the animals were orthotopically injected with 2 × 10^6^ Py230 cells in 50 μL of phosphate buffered saline (PBS) into the fourth mammary fat pad. The optimal Py230 cell number was determined using a pilot study in which mice were inoculated with a range of tumour cell concentrations: 5 × 10^4^; 1 × 10^5^; 1 × 10^6^; and 2 × 10^6^ cells per 50 μL PBS (Bao et al. 2015). The concentration of 2 × 10^6^ Py230 cells yielded the most consistent tumour growth over a 12 week period. Post‐injection, the animals were monitored twice per day for behavioural signs of pain, respiratory distress, and fur coat appearance throughout the study. The mice did not display any signs of pain and so analgesic drugs were not administered following orthotopic injections of Py230 cells. Animal welfare was monitored by the same researcher to ensure consistency. The body weight of mice was measured once a week and tumour volumes were measured twice per week, using a digital calliper, until they reached 50–70 mm^3^ (approx. 9 weeks post Py230 cell inoculation). Non‐tumour controls (*N* = 40) and tumour‐inoculated mice (*N* = 51) were arbitrarily assigned into either non‐stress standard housing conditions (*N* = 45) or morning restraint stress groups (*N* = 46) for the final 3 weeks of the study protocol. No formal randomisation procedure was conducted to allocate mice to experimental groups. This initially resulted in four different experimental groups, (1) a control group, Ctrl (*n* = 20); (2) a group that received restraint stress, RS (*n* = 20); (3) a cancer‐inoculated group, CAN (*n* = 25); and (4) a cancer‐inoculated group that received restraint stress, CRS (*n* = 26).

#### 2.1.3 Restraint Stress Paradigm

To induce and mimic psychological stress, half of the standard housed (RS) and tumour‐inoculated (CRS) mice were subjected to 3 weeks of daily inescapable restraint stress for 2 h each morning from 10.00 to 12.00. Animals were placed into a ventilated 50 mL conical tube where they could rotate from supine to prone positions, but not from head to tail (Buynitsky and Mostofsky 2009; Flint et al. 2005). The procedure is painless but produces a consistent non‐habituating spike in stress hormones. The animals' well‐being was continually monitored during the procedure.

#### 2.1.4 Study Endpoint

**FIGURE 1:**
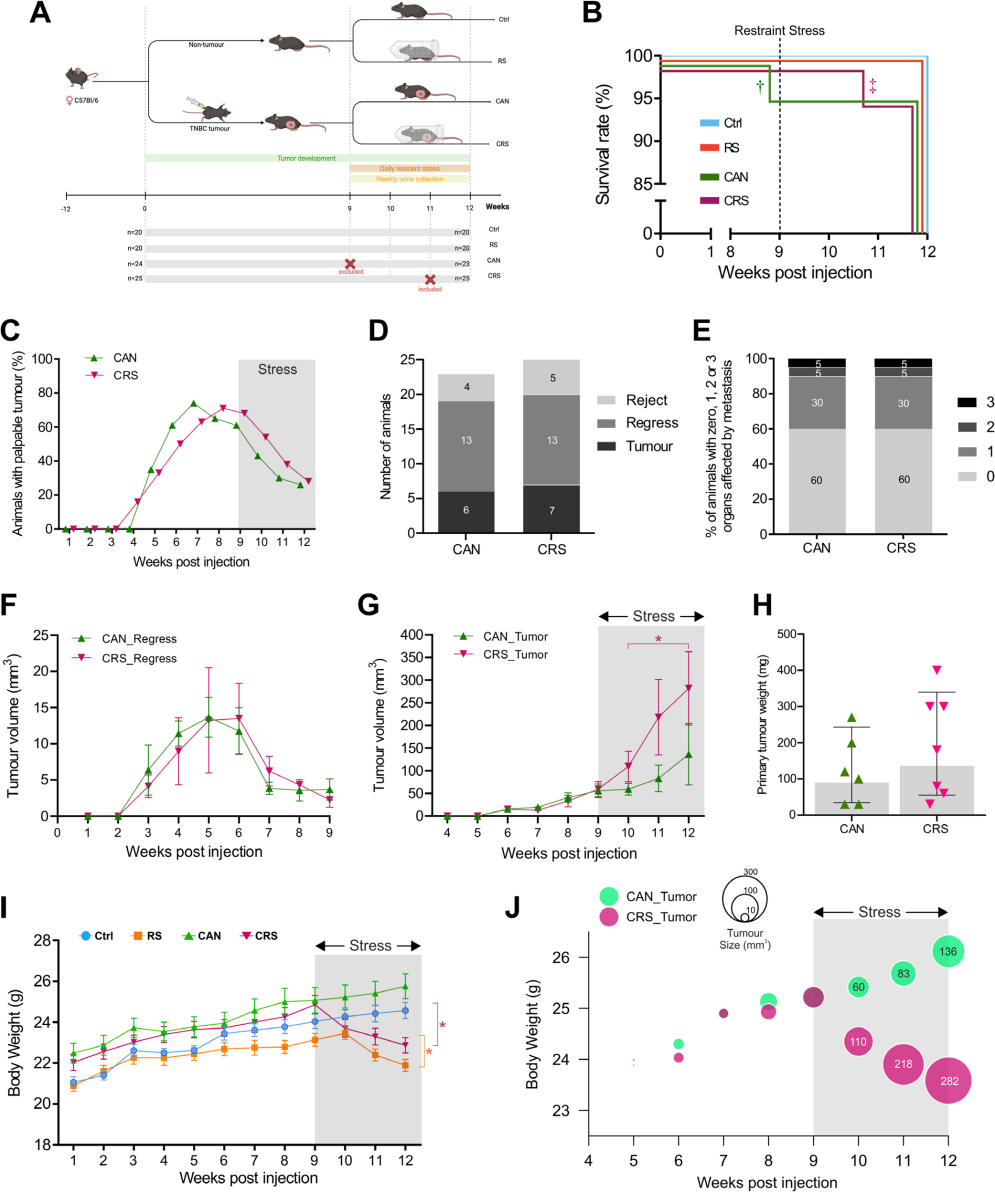
Restraint stress induces weight loss and increases tumour volume in a triple‐negative breast cancer mouse model. (A) Schematic overview of the four main groups of female mice and the 12‐week experimental timeline, including 3‐weeks of restraint stress from Weeks 9 to 12. (B) Kaplan–Meier curve showing the survival rates of each group of mice over the 12‐week experiment from 3 to 6‐months of age. Tumour cell inoculation and restraint stress had no significant impact on overall survival rates. In the CAN group one animal was found dead (†) and excluded from the study. In the CRS group one animal was sacrificed (‡) when the mean tumour diameter exceeded 1.2 cm and the brain was dissected and included in molecular/proteomic analyses. (C) Graph describing the tumour take rate in the CAN and CRS groups of mice. Tumours were undetectable until weeks 4–5 of the study. By Weeks 7–8, approximately 70% of the mice displayed a palpable mammary tumour. However, from week 9 onwards, approximately half of these tumours began to regress to undetectable levels by week 12 (the study endpoint). Stress did not appear to have a significant influence on tumour regression given that mice that were not restrained (CAN) also displayed similar levels of tumour regression. (D) Stacked bar graph showing the absolute numbers of mice in each group which displayed a palpable tumour at week 12 (CANTumor and CRSTumor), those mice that developed tumours which regressed by week 12 (CANRegress and CRSRegress), and mice that did not develop a detectable tumour throughout the 12‐week experimental period (CANReject and CRSReject). (E) Stacked bar graph showing the percentage of mice in the CAN and CRS groups that displayed secondary organ metastasis at the study endpoint. In both groups, 60% of mice did not display any detectable metastatic tumour growth, whereas 40% of the mice displayed either 1, 2 or 3 organs affected by tumour growth. (F) Graph showing tumour volumes in the CANRegress and CRSRegress groups of mice from week 1–9 post‐inoculation. Tumour volumes reached their peak at weeks 5–6 and then began to spontaneously regress (note this is before the restraint stress paradigm began). (G) Graph showing tumour volumes in the CANTumor and CRSTumor groups of mice from week 4–12 post‐inoculation. Both groups displayed similar size tumours at week 9, prior to the commencement of restraint stress in the CRS group (i.e., CAN 56 ± 15 mm^3^ Vs 58 ± 17 mm^3^ CRS). By week 12, however, tumours had grown in the CAN group to an average volume of 136 ± 63 mm^3^ and in the CRS group that received 3 weeks restraint stress to an average volume of 282 ± 81 mm^3^. Tumour volume was quite variable and so a Fisher's exact test for count data revealed that there was a significant difference in the number of tumours above 100 mm^3^ in the CRS versus CAN groups of mice after restraint stress had begun, that is, weeks 10–12 (*p*‐value = 0.0089; odds ratio = 7.0693). (H) After final size measurements were taken, tumours were weighed but there was no significant difference between the average tumour weights in the CAN and CRS groups (Student's *t*‐test, *t*(11) = 0.9861; *p* = 0.3453) (I) Graph showing the change in body weight over time in all four groups of mice. Mice were 3‐months of age at Week 1 and 6‐months of age at Week 12. There were gradual increases in body weight in the Ctrl and CAN groups. However, mice that received restraint stress from Weeks 9 to 12 showed a significant decrease in body weight at the study endpoint [two‐way ANOVA, F(11, 924) = 145.7; *p* = 0.0001]. The change in body weight for Ctrl mice from Week 9 to 12 was, on average, +0.54 g. The RS mice, however, lost on average − 1.26 g over the same time period. Similarly, the CAN group gained on average + 0.69 g, whereas the CRS group dropped −1.99 g from Week 9 to 12. (J) The mean body weights of mice in the CAN and CRS groups and their respective average tumour volumes were plotted over time, from Weeks 5–12, using a bubble plot. The size of each bubble corresponds to the average tumour volume. At Week 9, restraint stress began for the CRS group and the graph clearly shows a decrease in mean body weight and an increase in average tumour volume in the CRS group at Weeks 10–12. The CAN group of mice, on the contrary, continued to put on weight until the study endpoint. Ctrl, control; RS, restraint stress; CAN, cancer; CRS, cancer and stress; CANTumor, mice with sustained tumour growth; CRSTumor, stressed mice with sustained tumour growth; CANRegress, mice in which tumours regressed; CRSRegress, stressed mice in which tumours regressed; CANReject, mice that rejected cancer cells; and CRSReject, stressed mice that rejected cancer cells. Total number of animals (‘*n*’) in the four main groups, Ctrl *n* = 20; RS *n* = 20; CAN *n* = 23; CRS *n* = 25. Error bars represent ± 95% confidence intervals.

In both tumour‐inoculated groups (CAN and CRS), one animal was sacrificed for preliminary assessment of internal tumour growth. These two mice were excluded from further molecular or proteomic experiments. In addition, one mouse in the CAN group died between Weeks 8 and 9, most likely due to an intestinal tumour discovered post‐mortem. This animal was also excluded from the study. In the CRS group, one mouse was culled for welfare purposes at Week 11 when the mean diameter of its tumour exceeded 1.2 cm (Figure 1A). This animal was included in downstream molecular and proteomic experiments. All surviving tumour‐inoculated mice were then sacrificed at Week 12, after 3 weeks of standard group housing ± stress conditions. All animals in the Control (Ctrl) and Restraint Stress (RS) groups survived until the study's endpoint and were also culled at this same time‐point. The group sizes at the 12‐week study endpoint were as follows: Ctrl *n* = 20; RS *n* = 20; CAN *n* = 23; CRS *n* = 25 (Figure 1A,B). However, in both the CAN and CRS groups, tumour regression occurred in 13 mice. We named these groups CANRegress and CRSRegress. Mice that developed tumours which continued to grow for 12‐weeks were termed CANTumour and CRSTumour. In addition, four mice in the CAN group and five mice in the CRS group demonstrated complete cancer cell rejection and did not form any palpable tumour. These mice were referred to as CANReject and CRSReject. By splitting the CAN and CRS groups in such a way, this enabled us to investigate the differential impact of tumour growth, tumour regression, and cancer cell rejection on protein expression in the brain. Therefore, we were left with the following group numbers: Ctrl *n* = 20; RS *n* = 20; CANTumor *n* = 6; CANRegress *n* = 13; CANReject *n* = 4; CRSTumor *n* = 7; CRSRegress *n* = 13; CRSReject *n* = 5. We next decided to use one third of the mice (30 from 88) for subsequent behavioural studies (data not shown). Behaviour paradigms are known to influence protein expression in the brain and so these 30 mice were not included in the present study. The remaining two thirds (58) of behaviour‐naïve mice were used for molecular and proteomic analysis of brain tissue (data shown). The 30 mice removed from the present study consisted of *n* = 8 Ctrl, *n* = 8 RS, *n* = 7 CANRegress and *n* = 7 CRSRegress. This left us with final group sizes of Ctrl *n* = 12; RS *n* = 12; CANTumor *n* = 6; CANRegress *n* = 6; CANReject *n* = 4; CRSTumor *n* = 7; CRSRegress *n* = 6; CRSReject *n* = 5. Not all mice were utilised for every molecular study of brain tissue. The actual numbers used from each group are detailed in the respective figure legend.

#### 2.1.5 Tissue Collection

Culling was performed by schedule 1 approved methods and involved cervical dislocation followed immediately by decapitation and cessation of blood flow. Primary tumours were dissected, weighed, fixed in 10% neutral buffered formalin, and paraffin‐embedded for histological analysis. Lungs were also removed and fixed in 10% formalin. The left brain hemispheres were dissected into multiple brain structures, that is, the hippocampus, hypothalamus, prefrontal cortex, neocortex and cerebellum. The regions that remained were the midbrain, thalamus and striatum, which were grouped together. Each brain structure was flash frozen separately using liquid nitrogen. Furthermore, the spleens were collected in growth medium, that is, RPMI 1640 with 10% FBS, and mashed through a 40 μm cell strainer to acquire splenocytes. The splenocytes were concentrated by centrifugation at 300*g* for 10 min, resuspended in freezing medium (RPMI 1640 + 10% FBS + 10% DMSO) and stored in liquid nitrogen.

#### 2.1.6 Urine Collection

Urine samples were collected at four specified time points during the experiment to quantify corticosterone levels, that is, after 8 weeks of tumour development (Week 9), after 1 week of restraint stress (Week 10), after 2 weeks of restraint stress (Week 11), and after 3 weeks of restraint stress (Week 12). Spontaneously released urine was obtained by scruffing the mice and collected in sterile microcentrifuge tubes on Monday mornings, prior to initiating the restraint stress protocol. These samples were then stored at −80°C for subsequent analysis.

### 2.2 Urinary Corticosterone Assay

The quantitative analysis of urinary corticosterone concentrations at the four specified time points was carried out using the Corticosterone Parameter Assay Kit (KGE009, R&D Systems Inc., Minneapolis, USA), in accordance with the manufacturer's protocol. Optical density measurements were performed with a microplate reader (Asys UVM 340, Biochrom Ltd., Cambridge, UK). The mean minimal detectable dose (MDD) of corticosterone, as reported by the manufacturer, is 0.028 ng/mL.

### 2.3 Flow Cytometry

Flow cytometry was used to investigate the phenotypic profile of T‐cell populations (CD3^+^, CD4^+^ and CD8^+^) within the spleen. Splenocytes were defrosted and cultured using RPMI 1640 medium supplemented with 10% FBS, at a density of 1 × 10^6^ cells per well (in 12‐well plates), and maintained at 37°C and 5% CO_2_ for 24 h. Splenocytes were prepared as a single‐cell suspension of 1 × 10^6^ cells and resuspended in 100 μL FACS buffer (PBS + 1% BSA), followed by a 20‐min incubation at 4°C with the appropriate fluorescently‐labelled antibodies. Three different T‐cell markers were used: PE‐conjugated anti‐CD3ε (RRID: AB_312672), APC‐conjugated anti‐CD4 (RRID: AB_312696) and FITC‐conjugated anti‐CD8α (RRID: AB_312744). A 7‐amino‐actinomycin D (7‐AAD) dye was added to determine cell viability. T‐cell population data were acquired using a BD Accuri C6 Flow Cytometer (BD Biosciences, USA). Automatic colour compensation was conducted in FlowJo v.10.6.2 software (BD Biosciences, USA) using single‐staining controls for each fluorochrome. Initially, cells were gated for single live cells based on 7‐AAD exclusion, followed by gating CD3^+^ cells into CD4^+^ or CD8^+^ T‐cell populations.

### 2.4 Brain Cytokine ELISAs

Two distinct areas of the brain were investigated for cytokine expression, that is, levels of IL‐2, IL‐4 and IL‐10 were measured in the combined subcortical brain structures of the midbrain, thalamus and striatum, whereas the levels of TNF‐α, IFN‐γ and IL‐6 were measured in the prefrontal cortex. Frozen brain tissue samples were manually homogenised in 300 μL Tris buffer (100 mM Tris, 150 mM NaCl, 1 mM EDTA, 1% Triton‐X100 and 5% glycerol, pH 7.4) which contained phosphatase (PhosSTOP, Roche, UK) and protease inhibitors (cOmplete Mini, EDTA‐free, Roche, UK). The homogenate was agitated for 2 h at 4°C, followed by a microcentrifugation step (1300 rpm for 20 min at 4°C) where the supernatant was collected. Protein concentrations were determined using the DC Protein Assay (Bio‐Rad, UK) according to the manufacturer's instructions and the samples and standards were loaded in triplicates. DuoSet ELISA development kits (R&D Systems, Bio‐Techne, UK) for the cytokines TNF‐α (DY410), IL‐6 (DY406), IFN‐γ(DY485), IL‐4 (DY404), IL‐2 (DY402) and IL‐10 (DY417) were used according to the manufacturer's protocols. Optical densities were determined using a microplate reader (Asys UVM 340, Biochrom Ltd., Cambridge, UK) at 450 nm and wavelength correction 540 nm. The limit of detection (LOD) was variable for each cytokine (TNF‐α, IFN‐γ and IL‐10 = 31.2 pg/mL; IL‐6, IL‐4 and IL‐2 = 15.6 pg/mL). Data are presented as protein of interest in pg. per mg of total protein (pg/mg).

### 2.5 Ki67 Staining

Tumour and lung tissues embedded in paraffin were sectioned at 5 μm using a Leica RM2235 microtome (Leica Microsystems Ltd., UK). These sections were adhered to positively charged Superfrost Plus microscope slides (Thermo Fisher, UK). Slides were baked at 60°C overnight, followed by xylene‐mediated deparaffinisation and water‐mediated rehydration in decreasing ethanol concentrations. Slides were then transferred to 95°C antigen retrieval buffer (10 mM Tris, 1 mM EDTA, 0.05% TWEEN20, pH 9) for 20 min and then blocked in 5% BSA (Sigma Aldrich, UK) for 1 h. This was followed by overnight incubation (at 4°C) with a 1:400 dilution (in PBS and 2% BSA) of the primary antibody (Rabbit Ki‐67 [D3B5], Cell Signalling, RRID: AB_2687446). The slides were then washed and incubated with the pre‐adsorbed secondary antibody (Donkey anti‐Rabbit IgG H&L [Alexa Fluor488], Abcam, RRID: AB2571722), diluted 1:2000 in PBS and 2% BSA, for 4 h at 21°C. Nuclear counterstaining was carried out using DAPI (4′,6‐Diamidino‐2‐Phenylindole, Dihydrochloride, Sigma, UK) at a concentration of 10 ng/mL in H_2_O for 15 min. Finally, the slides were mounted using ProLong Gold antifade solution (Fisher Scientific, UK) and left to cure overnight. Staining was visualised with an upright confocal laser scanning microscope (Zeiss LSM 800 Axio Examiner.Z1, Germany) using a 40× magnification objective (Plan‐Apochromat objective 40×/1.1 W Corr M27, Zeiss, Germany). Imaging of the Ki67 staining (EXmax = 490 nm, EMmax = 525 nm) was performed with the 488 nm laser diode, while simultaneous detection of DAPI staining (EXmax = 350 nm, EMmax = 470 nm) was achieved using the 405 nm laser diode. The scanning resolution was set to 1024 × 1024 pixels, with the pinholes set to 1 airy unit (AU). Individual images were exported as TIFF files using ZEN software (Zeiss, Germany). CellProfiler 3.0.0 (http://cellprofiler.org), an open‐source Python‐based image analysis software, was used to quantify the percentage of Ki67‐positive nuclei. Briefly, nuclei were automatically detected using DAPI staining as a nuclear marker. The Ki67 index was quantified as the percentage of Ki67‐positive DAPI‐labelled nuclei. The following sequence of commands was employed: Import images > Convert Colour to Grey > Split Channels > Convert Blue RGB to ‘DAPI’ > Convert Green RGB to ‘Ki67’ > Identify Primary Objects (DAPI) > Identify Primary Objects (Ki67). Values above 20% are typically considered indicative of high‐grade proliferative activity (Ma et al. 2017).

### 2.6 Mass Spectrometry

#### 2.6.1 Sample Preparation

The proteomic profile of the whole hippocampus (cornu Ammonis and dentate gyrus) was determined using reversed phase nanoflow liquid chromatography mass spectrometry (nLC‐MS/MS). Each frozen hippocampus was finely minced and homogenised in lysis buffer (6 M Urea [Sigma Aldrich, UK], 2 M Thiourea [Sigma Aldrich, UK], 100 mM Tris–HCl [Sigma Aldrich, UK], 1% Benzonase Nuclease [Sigma Aldrich, UK] and protease/phosphatase inhibitors [cOmplete ULTRA tablets and PhosSTOP, Roche, Germany]) using a needle and syringe on ice. The homogenates were then centrifuged at 10000*g* for 10 min at 4°C. Supernatant protein concentration was determined using the DC Protein Assay (Bio‐Rad, UK) according to the manufacturer's protocol. Each hippocampus yielded 2–3 mg of total protein. A portion (1 mg) of each sample was diluted fourfold in ddH_2_O to ensure compatibility with the DC Protein Assay reagents (≤ 4 M Urea). Prior to protein digestion, 0.1 μg of bovine α‐Casein (C6780, Merck, UK) was added to each sample to act as an internal standard for relative quantification. Protein digestion was performed in solution by diluting the samples fivefold in 50 mM AmB (Ammonium Bicarbonate, Sigma Aldrich, UK) and 5 mM DTT (1,4‐Dithiothreitol, Sigma Aldrich, UK) at 20°C for 1 h. Next, IAA (Iodoacetamide, Sigma Aldrich, UK) was added to a final concentration of 20 mM. After 30 min in the dark, the IAA reaction was quenched by adding DTT to a final concentration of 5 mM. To further reduce the initial urea concentration to 1 mM, the solutions were diluted in 50 mM AmB. Trypsin (Thermo Fisher, UK) was then added at a 1:50 ratio (w/w) for 4 h at 37°C. Following tryptic cleavage, protein digestion was performed at 37°C overnight by adding more trypsin at a ratio (w/w) of 1:25. Acidifying the solution to pH < 3 using 5% TFA (Trifluoroacetic acid, Sigma Aldrich, UK) to a final concentration of 0.5% halted the trypsin reaction. Solid reversed phase extraction cartridges (Bond Elut C18, Agilent, UK) were used to desalt the digested hippocampal samples. Next, peptides were eluted using 60% acetonitrile/0.1% TFA. The samples were then concentrated using a speed‐vacuum centrifuge (SpeedVac SC100, Savant, UK) and resuspended in 5 μL 0.1% TFA [±1 μg/μL].

#### 2.6.2 Protein Data Acquisition

A volume of 5 μL of each hippocampal peptide sample (equivalent to 1 μg peptide digest) was analysed by nLC‐MS/MS (Dionex UltiMate 3000 RSLCnanoSystem, ThermoFisher) coupled to a hybrid quadrupole Orbitrap mass spectrometer (Q Exactive, ThermoFisher) equipped with a nanospray ion source (Nanospray Flex, ThermoFisher). A μ‐precolumn (300 μm I.D. × 5 mm C18 PepMap 100, 5 μm, 100 Å, ThermoFisher) was used to pre‐concentrate the peptides, set to a flow rate of 20 μL/min for 4 min using mobile phase A (0.1% (v/v) formic acid in hypergrade water, Merck KGaA). Subsequent separation was achieved by reversed phase on a nano column (Aclaim PepMap RSLC 75 μm × 15 cm nanoViper, ThermoFisher) at a flow rate of 200 nL/min using a gradient of mobile phase B (0.1% (v/v) formic acid in acetonitrile, LiChrosolv, Merck KGaA) from 1% to 30% B (curve 5) over 149 min, 30%–99% B (curve 6) over 10 min, held at 99% B for 5 min and back to 1% B over 1 min to equilibrate for 9 min, with a total chromatographic run time of 185 min. A stainless‐steel emitter (40 mm, “1/32” OD) was used post‐column. MS data were acquired in a data dependent manner; with full scan MS spectra (300–1650 m/z) recorded at a resolution of 140 000 at m/z 200 followed by the fragmentation of the top 10 most abundant precursor ions. Dynamic exclusion was set to 45 s, with charge exclusion set for unassigned and singly charged species. Automatic gain control (AGC) target was set to 1 × 10^6^ with a maximum injection time of 20 ms for full scan. Fragmentation of precursor ions was performed by higher‐energy collisional dissociation (HCD) with a normalised stepped collision energy of 20, 25 and 30, with a default charge state of 2. MS/MS scans were performed at a resolution of 17 500 at m/z 200 with an AGC target value of 1 × 10^5^ and a maximum injection time of 120 ms using an isolation window of 2.2 m/z. MaxQuant software (V.1.6.3.4, Max Planck Institute of Biochemistry) was used to process the acquired mass spectra against a SwissProt database (Proteome ID: UP000000589, *Mus Musculus* , accessed 09/2020) for protein identification. The searches were performed with the following settings: trypsin enzyme with up to two missed cleavages, phosphorylation (P) at serine, threonine and tyrosine residues as a variable modification, minimum peptide length was seven amino acids, maximum peptide mass was 4600 Da, protein false discovery rate (FDR) was set to 1% and minimum razor + unique peptides was set to 1 (minimum of 1 peptide for protein identification).

#### 2.6.3 Proteomic Data Analysis

For each of the 8 groups of mice, there were *n* = 3 peptide samples analysed by mass spectrometry. Each peptide sample consisted of a mixture of hippocampal tissue from a subset of mice in each group (with equal amounts of total protein per mouse), that is, *N* = 8 (Ctrl); *N* = 8 (RS); *N* = 6 (CANTumor); *N* = 6 (CANRegress); *N* = 4 (CANReject); *N* = 7 (CRSTumor); *N* = 6 (CRSRegress); *N* = 5 (CRSReject). The identified proteins, alongside their respective abundances (i.e., label‐free quantitation (LFQ) intensities), were exported as a ‘proteinGroups.txt’ file for further analysis. The list of identified proteins was filtered by removing potential contaminants (i.e., human proteins) and identified proteins with a charge < 2. Next, the obtained LFQ intensity data were further analysed using ProVision, an R‐shiny web‐based proteomics data analysis platform for downstream analysis of MaxQuant output data (Gallant et al. 2020). The data were log_2_ transformed and the median subtracted. Proteins were filtered based on a minimum of two identified values in at least one group. Missing values were imputed and the data were further analysed by calculating the fold change in normalised intensity between relevant groups. Proteins with a log_2_ fold change of ±0.5 and a *p* value < 0.05 were considered significantly altered (and *p*‐values were adjusted using the Benjamini‐Hochberg false discovery rate procedure). STRING version 12.0 was then used to perform functional enrichment analysis. A ranked list of all detected proteins for each pairwise comparison (i.e., upregulated proteins at the top of the list and downregulated proteins at the bottom) was used to identify biological functions that were overrepresented in the dataset. The ranked protein list was searched against the Gene Ontology (GO) databases for Biological Processes, Cellular Components, Molecular Functions, and KEGG (Kyoto Encyclopaedia of Genes and Genomes) Pathways. In addition, protein cluster analysis was performed in STRING version 12.0 using only the sets of proteins that were significantly up‐ or down‐regulated between groups and compared against the full background of 2300 proteins identified across all hippocampal protein samples. The unsupervised density‐based spatial clustering of applications with noise (DBSCAN) method (with an epsilon value of 12) was used to identify clusters of functionally‐related proteins that were up‐ or down‐regulated between groups.

### 2.7 Statistical Analysis

Due to the nature of the in vivo procedures (e.g., tumour cell inoculation) and the importance of maintaining animal welfare (e.g., during restraint stress protocols), the primary researcher was not blinded to the experimental groups. Moreover, no blinding was performed during the statistical analysis. No sample size calculation was performed a priori for this study. The optimal number of subjects per group was determined based on previous studies that had used the same cell line and mouse strain (Chung et al. 2015). We also based the sample sizes on our own previous work using in vivo mouse models of breast cancer (Flaherty et al. 2019). In both cases, sample sizes of 7–9 mice per group provided sufficient statistical power for detecting biologically meaningful effects. GraphPad Prism 8 software was used to perform the statistical analysis, which included two‐tailed Student *t*‐tests, one‐way ANOVAs with Holm‐Šídák's corrections for multiple comparisons, or two‐way ANOVAs with Holm‐Šídák post hoc corrections, as appropriate. The R statistical programming environment was used to conduct the Fisher's exact test for tumour volume count data.

No test for outliers was conducted for any of the datasets and no data points were excluded from statistical analysis. Data were assessed for normality using the Shapiro–Wilk test. Changes in body weight over time were normally distributed (Ctrl group W statistic = 0.9173, *p* = 0.2641; RS group W statistic = 0.9640, *p* = 0.8388; CAN group W statistic = 0.9505, *p* = 0.6450; CRS group W statistic = 0.9935, *p* = 0.9999). Tumour weights in the CAN and CRS groups were normally distributed (CAN group W statistic = 0.9159, *p* = 0.4760; CRS group W statistic = 0.9091, *p* = 0.3899). Corticosterone levels at 9 weeks in the control and tumour‐inoculated mice were normally distributed (Ctrl group W statistic = 0.8500, *p* = 0.0581; CAN group W statistic = 0.9353, *p* = 0.5016). At 12 weeks, ‘*n*’ numbers were too low to perform a Shapiro–Wilk test and so a Kolmogorov–Smirnov (KS) test was conducted along with visual inspection of the data points to assess normality. Corticosterone levels at 12 weeks were normally distributed (Ctrl group KS D statistic = 0.1958, *p* > 0.1000; RS group KS D statistic = 0.1947, *p* > 0.1000; CAN group KS D statistic = 0.2655, *p* > 0.1000; CRS group KS D statistic = 0.2211, *p* > 0.1000). To calculate changes in the ratio of CD4^+^/CD8^+^ T cells, the ‘*n*’ numbers in some of the groups were too low to test for normality and so Gaussian distributions were assumed based on a visual inspection of the data points and a parametric one‐way ANOVA was performed (*F* statistic = 5.324, *p* = 0.0007). Similarly, when analysing cytokine levels in the brains of each group of mice, ‘*n*’ numbers were too low to determine normality using a Shapiro–Wilk test and so the Kolmogorov–Smirnov test was used instead. The KS test results suggested data were normally distributed, except for the CANReject group (*n* = 4) in which the ‘*n*’ numbers were too low to generate D statistic values. Therefore, ELISA data were analysed using parametric one‐way ANOVAs. The mass spectrometry and proteomic data were log‐transformed and normalised by subtracting the median values prior to statistical tests. The data presented in bar charts and line charts represent the mean ± 95% confidence intervals (CI). Graphs were generated using either GraphPad Prism 8 software, the ggplot2 package in R programming environment, the online data visualisation tool SR Plot (Tang et al. 2023), or Adobe Illustrator software. The final figures were collated and generated using Corel Draw version 8.

## 3 Results

### 3.1 Stress‐Induced Weight Loss Correlates With Larger Tumor Volumes

Control (Ctrl) and restraint‐stressed (RS) mice underwent 12 weeks of standard group housing conditions and, in the final 3 weeks, the RS group experienced a daily 2‐h restraint stress paradigm. A third group of mice (CAN) were inoculated with Py230 tumour cells to establish an orthotopic syngeneic TNBC model. Finally, to mimic diagnosis‐associated psychological stress, a fourth group of tumour‐inoculated mice (CRS) received daily 2‐h restraint stress for a duration of 3 weeks (Figure 1A). The survival rates of all four groups were assessed at the scheduled study end‐point, that is, at 12 weeks post‐injection when the mice were 6 months of age. All mice in the Ctrl and RS groups survived the 12‐week protocol. However, one mouse from the CAN group died between Weeks 8–9, most likely due to an intestinal tumour which was found during the necropsy. This animal was excluded from the study. In the CRS group, one animal displayed a mean tumour diameter that exceeded 120 mm and was, therefore, sacrificed for welfare reasons between Weeks 10–11 (Figure 1B).

The percentages of tumour‐inoculated mice that developed palpable tumours peaked at Weeks 7–8 (Figure 1C) and were similar between the CAN and CRS groups (70%). Importantly, this time point came before the restraint stress paradigm began. Interestingly, approximately 9 weeks post‐tumour cell inoculation, spontaneous tumour regression began to occur in over half of the mice in both groups. The proportions of mice that developed a tumour (6/23 for CAN group and 7/25 for CRS group), the proportions in which the tumour regressed (13/23 for CAN group and 13/25 for CRS group), and those where no tumour developed at all (4/23 for CAN group and 5/25 for CRS group) were very similar between the two groups. Because tumour regression largely took place before Week 9, this suggested that the 3‐week stress paradigm (Weeks 9–12) had little or no effect on tumour regression (Figure 1D). Similarly, the proportions of mice that displayed visible secondary organ metastasis upon post‐mortem examination were identical between the CAN and CRS groups (Figure 1E), suggesting that restraint stress did not influence the metastatic potential of tumours in this Py230 breast cancer model. From then on, the CAN and CRS groups of mice were split into three distinct sub‐groupings: (1) mice that developed tumours which continued to grow for 12 weeks (CANTumor and CRSTumor), (2) mice that rejected the cancer cells and failed to develop a palpable tumour (CANReject and CRSReject), and finally, (3) mice that initially developed a small palpable tumour (by Weeks 4–7) which subsequently regressed by Week 9 (CANRegress and CRSRegress). This classification system is similar to a recent study by Patel et al. (2023) that showed tumour growth and spontaneous regression are regulated by CD4^+^ and CD8^+^ T‐cell responses. In our study, spontaneously regressed tumours displayed volumes of 13 mm^3^, on average, by Week 5 but then shrunk to below 5 mm^3^ by Week 9 (Figure 1F). More than half of the tumours that continued to develop, however, reached > 100 mm^3^ by Week 12. Interestingly, several tumours in the CRSTumor group of mice grew very large during the 3‐week restraint stress period. By Week twelve, 2/6 of the tumours in the CANTumor group were > 100 mm^3^, whereas 6/7 tumours in the CRSTumor group were > 100 mm^3^ (Figure 1G). Therefore, stress had a statistically significant effect on the number of tumours that exceeded 100 mm^3^ throughout the 3‐week restraint stress period (Fisher's exact test, *p* = 0.0089). However, there was no statistically significant difference in the weight of primary tumours excised at necropsy (Student's *t*‐test, *p* = 0.3453, Figure 1H). The 3‐week restraint stress paradigm also had a significant effect on the body weight of both tumour‐inoculated mice (CRS) and their respective controls (RS). On average, mice in the RS group lost 5% body weight and those in the CRS group lost 9% body weight throughout the restraint stress paradigm (i.e., Weeks 9 vs. 12). In contrast, mice in the Ctrl group and those in the CAN group gradually gained body weight throughout the duration of the study (Figure 1I). Therefore, chronic stress caused a decrease in weight, whereas cancer had no effect on body weight in this Py230 breast cancer mouse model. These results were also visualised by plotting the mean body weight against mean tumour volume from Weeks 5 to 12 for the CANTumor and CRSTumor groups (Figure 1J). Notably, the mean body weight of CRSTumor mice drops during the 3‐week restraint stress paradigm (25.2 ± 2.5 vs. 23.6 ± 1.9 g) while the mean tumour volume increases dramatically (59 ± 17 vs. 282 ± 81 mm^3^). The overall decrease in body weight due to the restraint stress paradigm correlated with larger tumour volumes in CRSTumor mice. Indeed, a significant negative correlation was observed between body weight and primary tumour volume in CRSTumor mice (Pearson's *r* = −0.578, *p* = 0.0004). In contrast, a non‐significant positive correlation was observed between body weight and primary tumour volume in CANTumor mice (Pearson's *r* = 0.929, *p* = 0.0668) (Figure 1J).

### 3.2 Mammary Tumour Growth Induces Systemic Elevations in Corticosterone, Which Are Further Augmented by Chronic Stress

**FIGURE 2:**
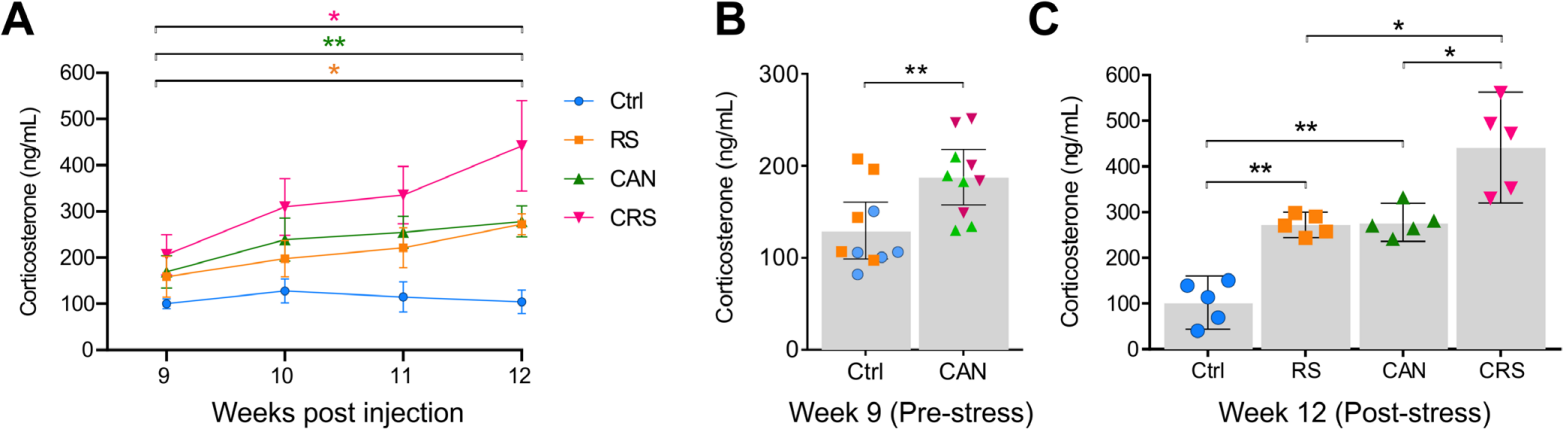
Mammary tumour cell inoculation alone is sufficient to increase urinary corticosterone levels over time. (A) Three weeks of sustained tumour growth and/or restraint stress significantly increased urinary levels of corticosterone in RS, CAN and CRS mice. Levels of urinary corticosterone remained stable in Ctrl mice over time (two‐way ANOVA *F*(9, 48) = 6.698; *p* = 0.0001, Holm‐Šídák *post hoc* tests). (B) Nine weeks post‐tumour cell inoculation, but before initiation of the restraint stress paradigm, urinary corticosterone levels were significantly higher in Py230 cell‐inoculated mice (CAN) compared to the Ctrl mice (Student's *t*‐test, *t*(18) = 3.038, *p* = 0.0071). (C) After 3 weeks of daily (2 h) restraint stress, urinary corticosterone levels were significantly increased in the stressed group (RS) compared to control (two‐way ANOVA, *F*(1, 16) = 41.45, Holm‐Šídák post hoc test, *p* = 0.0011). In the CAN group, 3 weeks of additional tumour development caused further increases to urinary corticosterone levels compared to control mice (two‐way ANOVA, *F*(1, 16) = 44.47, Holm‐Šídák post hoc test, *p* = 0.0010). Mice in the CRS group had significantly higher levels of urinary corticosterone compared to stressed only (RS) (Holm‐Šídák *post hoc* test, *p* = 0.0011) and tumour‐inoculated only (CAN) mice (Holm‐Šídák *post hoc* test, *p* = 0.0011). Data represent the means ± 95% confidence intervals. Abbreviations: Ctrl, control *n* = 5; RS, restraint stress *n* = 5; CAN, cancer *n* = 5; CRS, cancer and stress *n* = 5. ‘*n*’ = number of animals.

To confirm that the restraint stress paradigm induced a physiological stress response, urinary corticosterone levels were measured on a weekly basis, from Week 9 (i.e., after 9‐weeks of tumour development, but before the start of the restraint stress paradigm) until Week 12 (i.e., after 12‐weeks of tumour development and 3 weeks of daily restraint stress). The levels of corticosterone remained stable from Weeks 9 to 12 in Ctrl mice (100 ± 5 vs. 104 ± 12 ng/mL). However, 3 weeks of daily restraint stress significantly increased the levels of urinary corticosterone in RS mice (159 ± 20 vs. 272 ± 10 ng/mL; *p* = 0.0419, Figure 2A). Interestingly, at week 9 (i.e., pre‐stress) urinary corticosterone levels were significantly higher in tumour‐bearing mice compared to control mice (*p* = 0.0025, Figure 2B). This suggests that tumour growth alone can lead to raised levels of systemic glucocorticoids. Indeed, urinary corticosterone levels increased from Week 9 to 12 in the non‐stressed tumour‐inoculated mice (CAN group; 169 ± 20 vs. 278 ± 15 ng/mL; *p* = 0.0058, Figure 2A). Notably, corticosterone concentrations at Week 12 in the CAN group matched those measured in the restraint‐stressed (RS) mice (278 ± 15 vs. 272 ± 10 ng/mL, respectively), thus highlighting that peripheral tumour growth elicits a physiological stress response similar to an established psychological stress paradigm, such as 3 weeks of daily physical restraint. Moreover, corticosterone levels in the stressed cancer group (CRS) increased significantly from Weeks 9 to 12 (206 ± 19 vs. 442 ± 44 ng/mL; *p* = 0.0153, Figure 2A). In addition, urinary corticosterone levels were significantly higher in CRS mice compared to the control (*p* = 0.0041), RS (*p* = 0.0477) and CAN (*p* = 0.0477) groups of mice at Week 12 (two‐way ANOVA with Holm‐Šídák *post hoc* corrections for multiple comparisons, Figure 2C).

**FIGURE 3:**
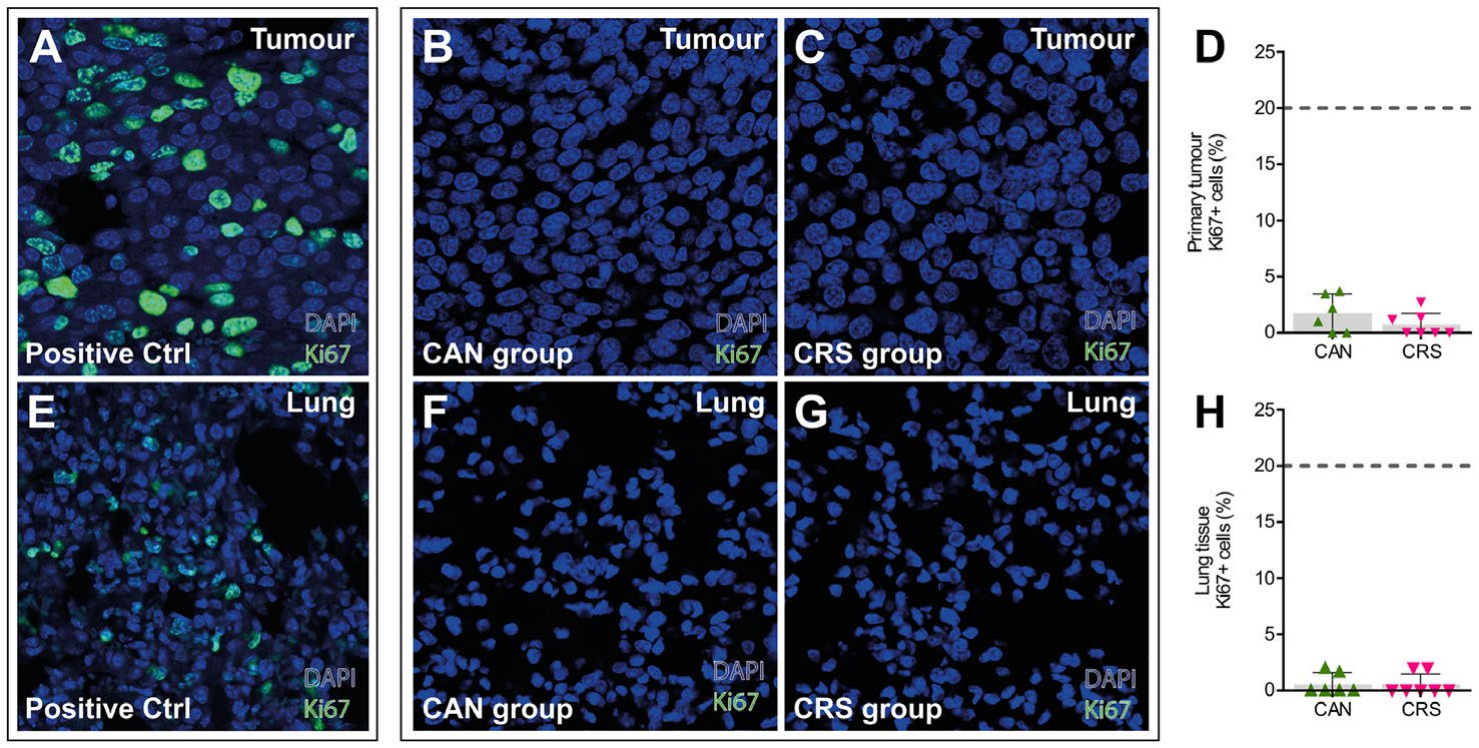
Py230 tumours display low levels of cellular proliferation and do not metastasize to the lungs. (A) Confocal image (40× magnification) of a 5 μm section from a highly proliferative Py230 tumour. This image shows an excised tumour from an aged mouse to demonstrate what a metastatic Py230 tumour should look like when immunofluorescently stained for the cell proliferation marker Ki67 (green) and the nuclear stain DAPI (blue) (i.e., a positive control for antibody staining). (B, C) Excised and 5 μm sectioned mammary tumours from the CAN and CRS groups of mice stained for DAPI nuclei (blue) and Ki67 (green). (D) The percentages of tumour cell nuclei that stained positive for Ki67 were below 5% in all mice and well below the 20% threshold that would suggest the tumour is likely to be metastatic. (E) Confocal image (40× magnification) of a 5 μm section of lung tissue from an aged Py230 tumour‐bearing mouse. This image demonstrates metastatic Py230 tumour cell invasion into the lungs. Py230 cells stain positive for the proliferation marker Ki67 (green) and cell nuclei stain positive for DAPI (blue). (F, G) Excised and sectioned lung tissue from the CAN and CRS groups of mice stained for Ki67 (green) and DAPI nuclei (blue). (H) The percentages of tumour cell nuclei that stained positive for Ki67 were below 3% in all mice and well below the 20% threshold that would suggest the primary tumours have metastasized to the lungs. CAN, cancer *n* = 6; CRS, cancer and stress *n* = 7. ‘*n*’ = number of animals. Data represent the means ± 95% confidence intervals.

Although the systemic concentrations of corticosterone were raised in CRS mice, the 3‐week restraint stress paradigm did not appear to increase the metastatic potential of the primary tumour. Post‐mortem immunofluorescence for Ki67 (a marker of cell proliferation) was performed on tissue sections from the primary tumour and from the lungs of mice belonging to the CANTumor and CRSTumor groups (Figure 3). The primary tumour showed low levels of proliferation (< 5% of cells stained for Ki67) in both CAN and CRS groups (Figure 3B–D). Similarly, the lungs showed even lower levels of positive staining for Ki67 (Figure 3F–H), suggesting that the tumours generated by Py230 breast cancer cells are not aggressive nor metastatic in our 6‐month‐old immunocompetent mouse cohort (Steenbrugge et al. 2019).

### 3.3 Tumour‐Induced Elevation in Splenic T Cell Ratio (CD4 ^+^/CD8 ^+^) is Inhibited by Chronic Stress

**FIGURE 4:**
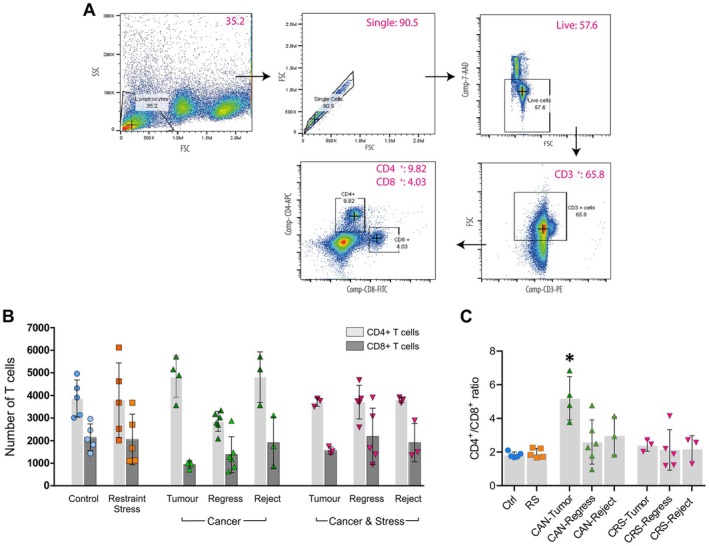
Non‐stressed mice that developed tumours display differences in splenic CD4^+^ and CD8^+^ effector T cell ratios. (A) Splenic T cell population numbers were assessed using flow cytometry as represented by the gating scheme. Live CD3^+^ T cells were separated into CD4^+^ and CD8^+^ T cell populations and counted. (B) Bar graph showing the raw numbers of CD4^+^ and CD8^+^ T cells from the spleens of each group of mice. (C) Bar graph showing the ratio of CD4^+^/CD8^+^ T cells in the spleens of each group of mice. There was an increase in the ratio of CD4^+^ to CD8^+^ T cells in non‐stressed mice in which tumours fully developed (CAN‐Tumour) (one‐way ANOVA, *F*(7, 26) = 5.324, *p* = 0.0007 with Holm‐Šídák's multiple comparisons *post hoc* tests). Data represent the means ± 95% confidence intervals. Abbreviations and ‘*n*’ numbers; ‘*n*’ = number of animals: Ctrl, control (*n* = 5); RS, restraint stress (*n* = 5); CAN‐Tumour, mice with sustained tumour growth (*n* = 4); CAN‐Regress, mice in which tumours regressed (*n* = 6); CAN‐Reject, mice that rejected cancer cells (*n* = 3); CRS‐Tumour, stressed mice with sustained tumour growth (*n* = 3); CRS‐Regress, stressed mice in which tumours regressed (*n* = 5); CRS‐Reject, stressed mice that rejected cancer cells (*n* = 3).

It is well established that the adaptive immune response determines one's ability to fight tumour growth. T cells, especially cytotoxic CD8^+^ T cells, serve an important role in killing tumour cells (Sánchez‐Paulete et al. 2017). As many of the Py230 primary tumours in our immune competent mouse model underwent spontaneous regression or failed to develop at all after cancer cell inoculation, the immune effector populations of CD4^+^ and CD8^+^ T cells from the spleen were examined using flow cytometry (Figure 4A). As before, groups were divided into animals that displayed sustained tumour development until the scheduled endpoint (CANTumor & CRSTumor), animals in which the tumour initially developed but underwent spontaneous regression (CANRegress & CRSRegress) and animals in which Py230 cancer cells were rejected (CANReject & CRSReject). No statistical differences were observed in the raw numbers of CD4^+^ or CD8^+^ T cells in the spleens of non‐stressed (CAN) and stressed (CRS) tumour‐inoculated mice (Figure 4B). However, the ratio of CD4^+^ to CD8^+^ positive T cells in non‐stressed tumour‐developed (CANTumor) mice was significantly higher compared to all other groups (one‐way ANOVA, *p < 0.05, Figure 4C). Interestingly, 3 weeks of restraint stress in the CRSTumor group appeared to prevent any increase in the CD4^+^ to CD8^+^ T cell ratio when compared to the CANTumor group (*p* = 0.0169, Figure 4C). These results suggested a significant divergence in immune regulation and T cell numbers in stressed versus non‐stressed tumour‐bearing mice. Moreover, the observation that CANTumor mice had significantly higher CD4^+^/CD8^+^ T cell ratios compared to CANRegress and CANReject groups suggested underlying differences in immune functioning and susceptibility to tumour development and maintenance (Figure 4C).

### 3.4 Tumour Inoculation Elevates Proinflammatory TNF‐α Expression and Dampens the Levels of Anti‐Inflammatory IL‐10 Cytokines in the Brain

**FIGURE 5:**
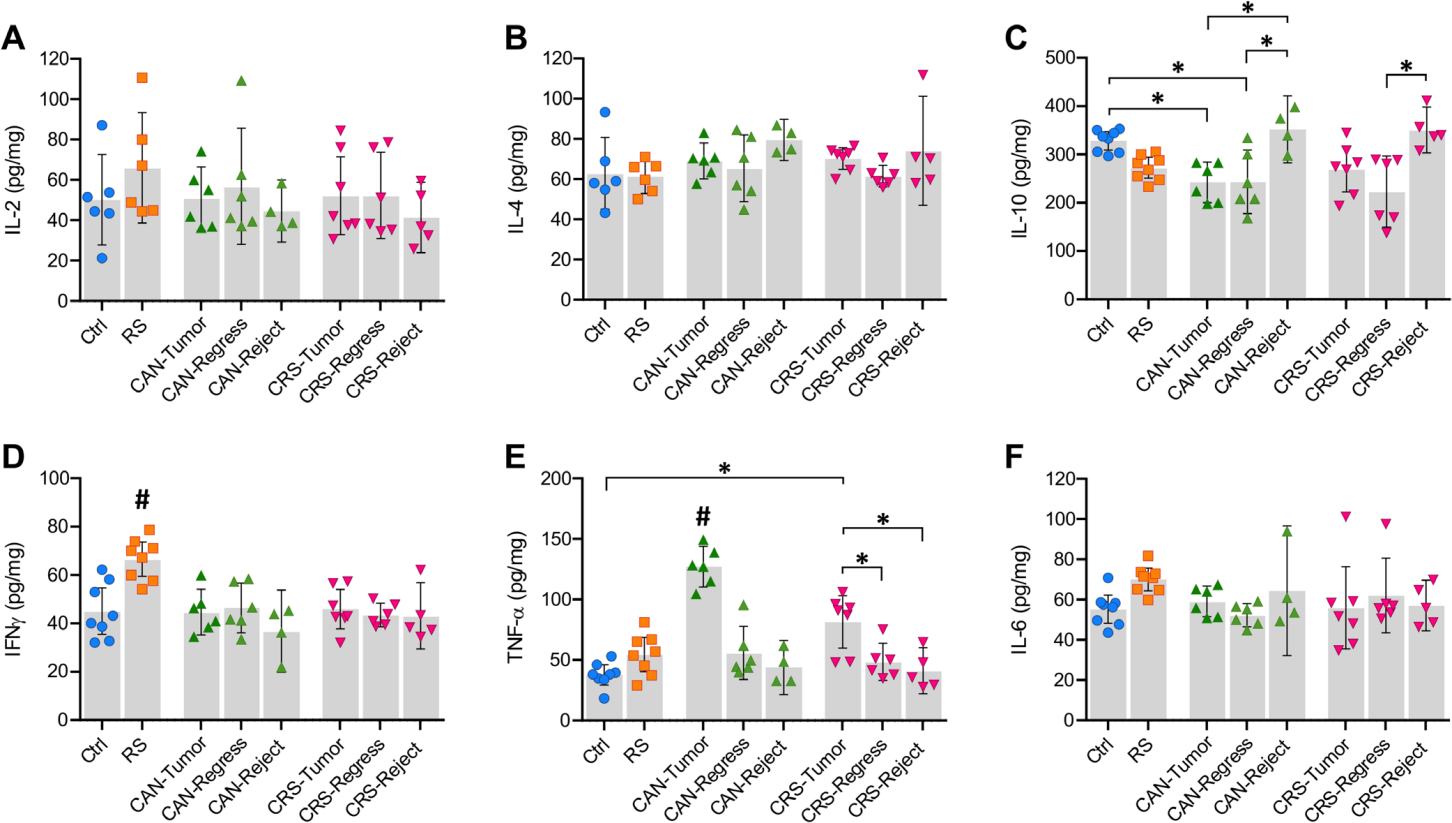
Differential cytokine expression profiles observed in the brains of stressed and/or tumour‐inoculated mice. (A, B) There were no differences in the levels of interleukin‐2 (IL‐2) and interleukin‐4 (IL‐4) in brain tissue samples composed of the midbrain, thalamus and striatum structures. (C) However, the anti‐inflammatory cytokine, interleukin‐10 (IL‐10), was down‐regulated in the midbrain, thalamus and striatum of mice in which Py230 mammary tumours developed (CAN‐Tumour) or spontaneously regressed (CAN‐Regress) (one‐way ANOVA, *F*(7, 42) = 6.884, *p* = 0.0001). In contrast, IL‐10 levels remained at or above control levels in the brains of mice in which tumours did not develop to begin with, that is, CAN‐Reject and CRS‐Reject. (D–F) The proinflammatory cytokines interferon‐ɣ (IFNɣ), tumour necrosis factor‐α (TNF‐α), and interleukin‐6 (IL‐6) were measured in brain tissue from the prefrontal cortex. (D) IFNɣ was upregulated (#) in the prefrontal cortex of non‐tumour stressed mice (RS) following 3 weeks of daily restraint when compared to all other groups (one‐way ANOVA, F(7, 42) = 5.940, *p* = 0.0001). (E) TNF‐α levels were significantly higher (#) in the prefrontal cortex of mice that displayed sustained mammary tumour growth (CAN‐Tumour) compared to all other groups (one‐way ANOVA, *F*(7, 42) = 18.90, *p* = 0.0001). As such, TNF‐α expression was significantly lower in CRS‐tumour mice that also received 3 weeks of restraint stress compared to CAN‐Tumour mice (*p* = 0.0004). However, TNF‐α expression remained higher in CRS‐Tumour mice versus Ctrl (*p* = 0.0003), CRS‐Regress (*p* = 0.0211) and CRS‐Reject (*p* = 0.0042) groups, suggesting that sustained tumour growth significantly increases TNF‐α in the prefrontal cortex of the brain. (F) There were no differences in the level of IL‐6 in the prefrontal cortex between any of the groups. Statistical analyses were performed using one‐way ANOVAs and Holm‐Šídák post hoc tests. Data represent the mean ± 95% confidence intervals. Abbreviations and ‘*n*’ numbers; ‘*n*’ = number of animals: Ctrl, control (*n* = 8); RS, restraint stress (*n* = 8); CAN‐Tumour, mice with sustained tumour growth (*n* = 6); CAN‐Regress, mice in which tumours regressed (*n* = 6); CAN‐Reject, mice that rejected cancer cells (*n* = 4); CRS‐Tumour, stressed mice with sustained tumour growth (*n* = 7); CRS‐Regress, stressed mice in which tumours regressed (*n* = 6); CRS‐Reject, stressed mice that rejected cancer cells (*n* = 5).

To investigate the impact of breast cancer on the neuroinflammatory state of the brain, cytokine levels were measured in different areas of the mouse brain. The concentrations of interleukin‐2 (IL‐2) and IL‐4 were analysed in tissue homogenate composed of several major sub‐cortical brain areas, specifically the midbrain, thalamus and striatum regions. Neither stress nor cancer caused any significant changes in IL‐2 or IL‐4 concentrations in the mouse brain (Figure 5A,B). However, changes in the levels of IL‐10 in response to tumour growth were evident (Figure 5C). IL‐10 is a potent anti‐inflammatory cytokine, which can suppress inflammation via inhibition of IL‐1β, IL‐6, IL‐8, IL‐12 and TNF‐α secretion (Sochocka et al. 2017). In breast cancer, IL‐10 is involved in the anti‐tumour response via inhibition of gene expression, cytokine synthesis by T cells and macrophages and modulation of their antigen presentation function (Esquivel‐Velázquez et al. 2015). A significant decrease in IL‐10 expression was observed in the brains of Py230 cell‐inoculated mice that developed tumours (CANTumor; *p* = 0.024) or whose primary tumour regressed (CANRegress; *p* = 0.028) in comparison to control mice (one‐way ANOVA, *F*(7, 42) = 6.884, *p* = 0.0001, Figure 5C). Interestingly, significantly higher levels of IL‐10 were observed in the brains of mice in which cancer cells were rejected (CANReject) when compared to CANTumor (*p* = 0.013) or CANRegress groups (*p* = 0.014) (Figure 5C). The CRSReject group also displayed higher brain IL‐10 concentrations than the CRSRegress mice (*p* = 0.001) (Figure 5C). There was no evidence that the restraint stress had any effect on brain IL‐10 levels. These results suggested that growth of the mammary tumour over time leads to dampened levels of the anti‐inflammatory cytokine, IL‐10, in subcortical brain structures.

Next, the levels of three pro‐inflammatory cytokines were measured in tissue homogenate dissected specifically from the prefrontal cortex, an area of the brain important for working memory and higher cognitive functions (Funahashi 2017). IFNɣ is often produced in the periphery by activated T cells and NK cells in response to tumour growth (Burgos‐Panadero et al. 2019). The levels of IFNɣ in the prefrontal cortex of mice did not change in the CAN or CRS groups in response to tumour growth. However, 3 weeks of daily restraint stress significantly increased IFNɣ concentrations in the prefrontal cortex of RS mice (one‐way ANOVA, *F*(7, 42) = 5.940, *p* = 0.0001, Figure 5D). Interestingly, tumour cell inoculation prevented this stress‐mediated increase in IFNɣ in the prefrontal cortex of all CRS mice. Restraint stress had little or no effect on the levels of TNF‐α expression in the prefrontal cortex of control or cancer mice, although TNF‐α expression was lower in CRSTumor compared to CANTumor samples (*p* = 0.0004, Figure 5E), suggesting that stress may slightly attenuate cancer‐induced increases in TNF‐α expression. Importantly, TNF‐α concentrations only increased in mice that displayed robust tumour growth and not in those mice in which tumours regressed or did not develop at all in the first place. This strongly suggested that increases in TNF‐α in the prefrontal cortex were a consequence of active breast cancer and sustained tumour growth. Finally, there were no significant changes in the levels of IL‐6 cytokine in the prefrontal cortex of either stressed or tumour‐bearing mice (Figure 5F).

### 3.5 Sustained Mammary Tumour Growth Downregulates Proteins Involved in Mitochondrial Respiration in the Hippocampus

**FIGURE 6:**
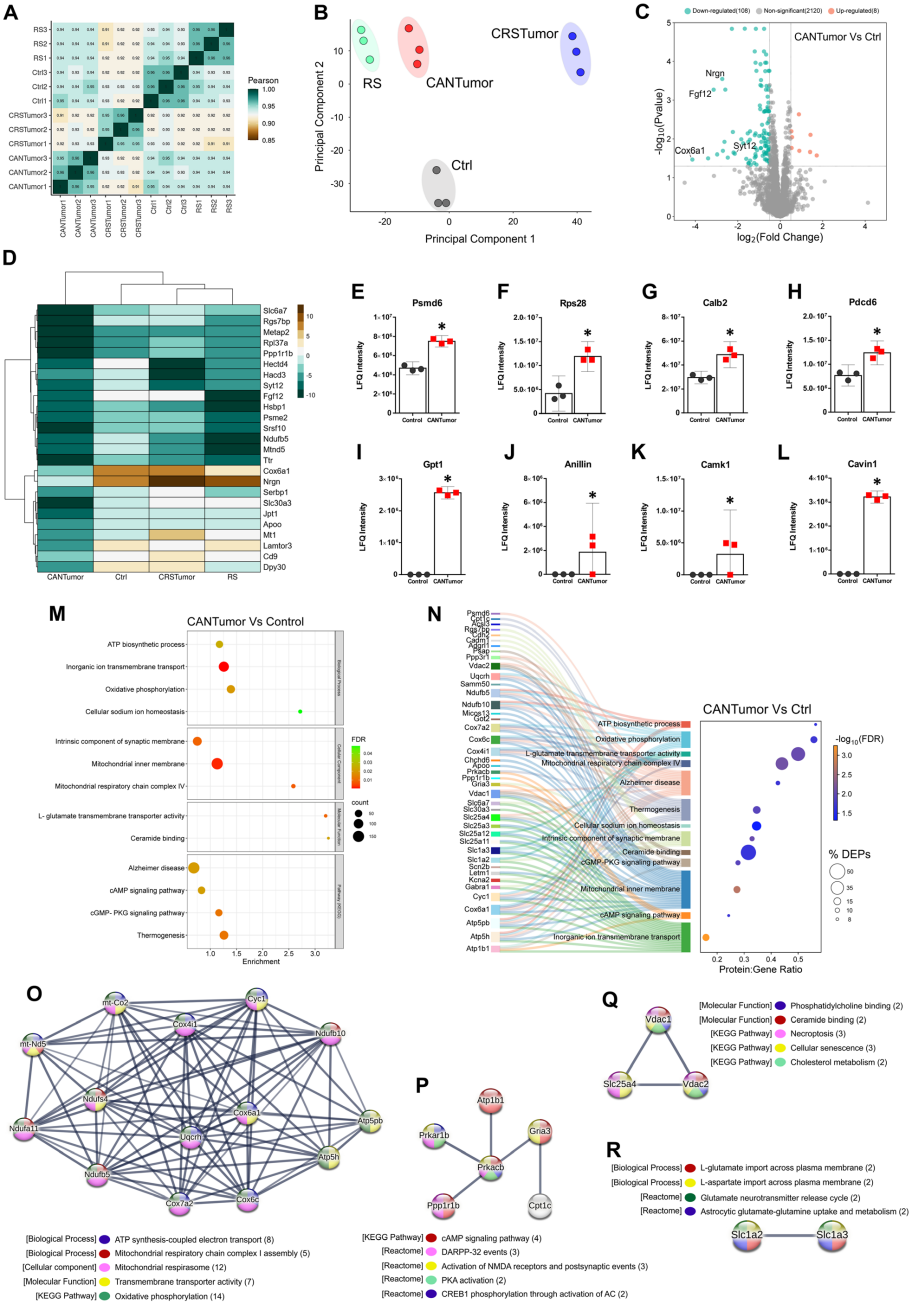
Sustained mammary tumour growth downregulates proteins involved in hippocampal synaptic plasticity. (A) Correlation matrix displaying the Pearson *r* values for each protein sample pairwise comparison between Ctrl, RS, CANTumor and CRSTumor groups of mice. Shades of green represent higher Pearson correlation scores, whereas shades of brown depict less correlated comparisons. (B) Principal component analysis (PCA) of the key features in the mass spectrometry data sets that best describe the variance between Ctrl, RS, CANTumor and CRSTumor hippocampal proteomes. (C) Volcano plot depicting the up‐ and down‐regulated proteins in the CANTumor protein samples compared to the Ctrl group. A total of 108 proteins (green dots) were down‐regulated and 8 up‐regulated (orange dots). Proteins expressing a log_2_ fold change > ±0.5 and a *p*‐value < 0.05 (using the Benjamini‐Hochberg false discovery rate correction) were considered significant. (D) Clustered heatmap of the top 25 significantly downregulated proteins in the CANTumor group compared to Ctrl samples. The relative expression of each protein in the RS and CRSTumor samples is also shown. The clustering method used was Ward.D which utilises the sum of squared distances to minimise the merging of clusters. (E–L) All 8 up‐regulated proteins in the CANTumor group compared to Ctrl samples. Displayed on the *Y*‐axes of the bar charts are the raw label‐free quantitation (LFQ) intensity values for each protein. Data represent the means ± 95% confidence intervals. (M) Gene ontology (GO) enrichment analysis was performed in STRING v.12.0 using a ranked list of all proteins detected in the CANTumor and Ctrl groups of mice. Proteins upregulated in CANTumor hippocampus ranked at the top of the list and downregulated proteins ranked at the bottom. Significantly enriched biological processes (BP), cellular components (CC), molecular functions (MF) and KEGG (Kyoto Encyclopaedia of Genes and Genomes) pathways are displayed against the enrichment score, along with the number of proteins (count) detected in our dataset which are linked to each GO term. The colour of each dot corresponds to its false discovery rate (FDR) value. (N) Differentially expressed proteins (DEPs) associated with enriched GO terms are listed to the left of the Sankey plot and connected to one or more biological process, cellular component, molecular function or KEGG pathway to the right. The Protein:Gene Ratio (*X*‐axis) represents the proportion of proteins associated with that particular GO term which were detected in our dataset. The bubble size depicts the percentage of detected proteins linked to each GO term that showed differential expression, that is, (no. of DEPs/no. of detected proteins associated with that GO term) × 100. The bubble colour represents −log_10_(FDR) value. (O–R) Clusters of functionally‐related proteins that were significantly down‐regulated in the CANTumor versus Ctrl group. The colours of each protein represent their functions with reference to the associated BPs, CCs, MFs, KEGG pathways or Reactome pathways. Ctrl, control; RS, restraint stress; CANTumor, mice with sustained tumour growth; CRSTumor, stressed mice with sustained tumour growth.

The hippocampus plays an important role in learning and memory formation and is particularly vulnerable to physiological and psychological stressors due to its high density of glucocorticoid and mineralocorticoid receptors (Kim et al. 2015). To further study the effects of cancer and stress on the brain, a shotgun proteomic analysis of the hippocampus was conducted using nLC‐MS/MS. In total, 2300 valid proteins were identified between all groups. A correlation matrix was generated to visualise the extent to which protein sample replicates were correlated (Figure 6A). Within‐group protein sample replicates were highly correlated (Pearson *r* ≥ 0.95). CRSTumor sample replicates were the least correlated to other groups. This was confirmed using principal component analysis (PCA) which describes how the CRSTumor samples (in blue) are further separated from the other three mouse groups along the PC1 axis (Figure 6B). Next, a volcano plot was generated to analyse the proteins that were significantly up‐ or down‐regulated in the CANTumor group compared to the control mice. 108 proteins were found to be downregulated by sustained tumour growth, whereas only 8 proteins were upregulated (Figure 6C). A clustered heatmap displaying the top 25 down‐regulated proteins in the CANTumor versus Ctrl group also illustrates the relative abundance of each protein in the restraint stress (RS) mice and in animals subjected to both cancer and stress (CRSTumor) (Figure 6D). Eight proteins were upregulated in the hippocampus in response to sustained tumour growth, including Psmd6, Rps28, calretinin (Calb2) and Pdcd6 (Figure 6E–H). Interestingly, four proteins that were upregulated by cancer were not detectable at all in the control (Ctrl) hippocampal protein samples, that is, Gpt1, Anillin, Camk1 and Cavin1 (Figure 6I–L). Next, gene ontology (GO) enrichment analysis was performed using STRING version 12.0, in which a ranked list of all proteins detected between the CANTumor and Ctrl groups (2236) were ordered such that the up‐regulated proteins ranked at the top and down‐regulated proteins at the bottom of the list. Significantly enriched (FDR < 0.05) biological processes (BP), cellular components (CC), molecular functions (MF) and KEGG pathways were then ordered based on their enrichment scores (Figure 6M). Proteins that are known to be involved in (1) the mitochondrial respiratory chain complex, (2) ATP synthesis, (3) oxidative phosphorylation, (4) cAMP‐dependent kinase activity and (5) lipid metabolism were differentially expressed in the hippocampus of CANTumor versus Ctrl mice. KEGG pathway analysis suggested that cellular processes akin to those seen in Alzheimer's disease brains may be triggered in the hippocampus in response to sustained tumour growth (Figure 6M). Next, a Sankey plot was generated to display the protein: gene ratio for each of the GO terms identified by the functional enrichment analysis (Figure 6N). The protein: gene ratio is the proportion of genes that make up the full GO term list, which had a corresponding protein match in our mass spectrometry dataset. Listed on the left‐hand side of the Sankey plot are all the differentially expressed proteins (DEPs) associated with each of the functionally enriched GO terms. STRING v.12.0 was also used to identify functionally related clusters of DEPs. Upregulated and downregulated sets of proteins were inputted separately into STRING, and functional enrichment analysis was performed against the full background of 2300 identified proteins in our mass spectrometry dataset. There were no functionally related clusters of upregulated proteins. However, 4 sets of protein clusters were identified in the downregulated set of DEPs (Figure 6O–R). The largest cluster of 14 proteins are involved in mitochondrial respiration and ATP synthesis (Figure 6O). A cluster of 6 proteins known to be involved in cAMP‐dependent kinase cellular signalling events and CREB1 phosphorylation was also identified (Figure 6P). A cluster of 3 DEPs involved in lipid metabolism, necroptosis, and cellular senescence was also downregulated (Figure 6Q). Finally, two proteins, Slc1a2 and Slc1a3, known to be involved in glutamate release and recycling across cellular membranes, were downregulated in the hippocampus of CANTumor versus Ctrl mice.

### 3.6 Restraint Stress Downregulates Mitochondrial, Ribosomal and Proteosomal Protein Clusters in the Hippocampus

**FIGURE 7:**
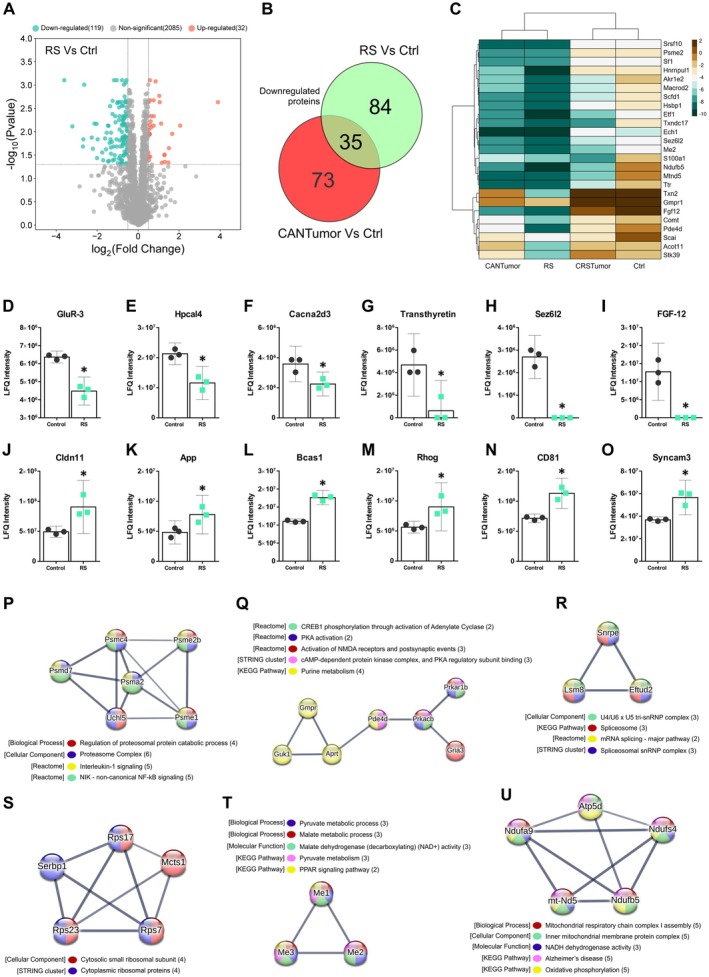
Chronic daily restraint stress downregulates a distinct set of hippocampal proteins compared to sustained tumour growth. (A) Volcano plot depicting the up‐ and down‐regulated proteins in the RS group compared to Ctrl group. A total of 119 proteins (green dots) were downregulated and 32 up‐regulated (orange dots). Proteins expressing a log_2_ fold change > ±0.5 and a *p*‐value < 0.05, using the Benjamini‐Hochberg false discovery rate (FDR) correction, were considered significant. (B) Venn diagram detailing the number of downregulated proteins in common between the RS versus Ctrl and the CANTumor veersus Ctrl comparisons. (C) Clustered heatmap of the top 25 significantly downregulated proteins in the RS group compared to Ctrl samples. The relative expression of each protein in the CANTumor and CRSTumor samples is also shown. The Ward.D clustering method was used which utilises the sum of squared distances to minimise the merging of clusters. (D–I) A selection of functionally interesting downregulated proteins in the RS group compared to Ctrl samples. Displayed on the *Y*‐axes of the bar charts are the raw label‐free quantitation (LFQ) intensity values for each protein. (J–O) A selection of functionally interesting upregulated proteins in the RS group compared to Ctrl samples. Data represent the means ±95% confidence intervals. (P–U) Clusters of functionally‐related proteins that were significantly downregulated in the RS versus Ctrl group. The colours of each protein represent their functions with reference to the associated biological processes (BP), cellular components (CC), molecular functions (MF), KEGG (Kyoto Encyclopaedia of Genes and Genomes) pathways, STRING clusters or Reactome pathways. Ctrl, control; RS, restraint stress; CANTumor, mice with sustained tumour growth; CRSTumor, stressed mice with sustained tumour growth.

Protein abundances in the restraint stress (RS) group of mice appeared to be most closely correlated with the CANTumor group (Figure 6A,B). Analysing the differentially regulated proteins in the RS versus Ctrl group revealed 119 downregulated proteins and 32 up‐regulated proteins (Figure 7A). A total of 35 of the downregulated proteins in the RS group were also downregulated in the CANTumor group when compared to the Ctrl samples (Figure 7B). However, 84 of the proteins downregulated in response to stress were unaffected by cancer. Therefore, the pathophysiological stress elicited by tumour growth impacts the hippocampal proteome in a different manner to the psychological stress of daily physical restraint. A clustered heatmap displaying the top 25 down‐regulated proteins in the RS versus Ctrl group was generated to also illustrate the relative abundance of each protein in the tumour‐bearing (CANTumor) mice and in animals subjected to both cancer and stress (CRSTumor) (Figure 7C). Six of the most notable proteins downregulated by restraint stress were GluR3 (Gria3), hippocalcin‐like protein 4 (Hpcal4), voltage‐dependent calcium channel (Cacna2d3), transthyretin (Ttr), seizure 6‐like protein 2 (Sez6l2) and fibroblast growth factor 12 (FGF‐12) (Figure 7D–I). Proteins of interest that displayed significant upregulations in response to restraint stress included claudin‐11, amyloid precursor protein (App), brain enriched myelin associated protein 1 (Bcas1), Rhog, CD81 and Syncam3 (Figure 7J–O). Gene ontology (GO) enrichment analysis was performed using STRING v.12.0 in which a ranked list of all proteins detected between the RS and Ctrl groups (2236) were ordered such that the upregulated proteins ranked at the top and downregulated proteins at the bottom of the list. There were no statistically significant GO term enrichments found between RS and Ctrl groups.

STRING v.12.0 was then used to identify functionally‐related clusters of DEPs. Up‐ and down‐regulated sets of proteins were input separately into STRING, and functional enrichment analysis was performed against the full background of 2300 identified proteins in our mass spectrometry dataset. There were no functionally‐related clusters within the set of 32 upregulated proteins. However, within the set of 119 downregulated proteins, there were 6 protein clusters that displayed 3 or more members (Figure 7P–U). A cluster of 6 DEPs is proteosome‐related proteins and may be involved in regulating the NF‐κB signalling pathway (Figure 7P). The largest cluster of 7 proteins are known to be involved in synaptic AMPA and NMDA receptor activation events, leading to cAMP‐dependent protein kinase A signalling and CREB1 phosphorylation in neurons (Figure 7Q). A cluster of 3 DEPs is known to be involved in regulating mRNA splicing events in the cell (Figure 7R). There was also a cluster of 5 cytoplasmic ribosomal proteins downregulated in RS vs. Ctrl samples (Figure 7S). The NADP‐dependent malic enzymes, Me1, Me2 and Me3 were also downregulated in the hippocampus of RS mice, suggesting dysregulations in malate metabolic processes and PPAR signalling events (Figure 7T). Finally, a mitochondria‐associated cluster of 5 proteins was downregulated in RS versus Ctrl mice (Figure 7U) suggesting disruptions to the mitochondrial respiratory chain complex and oxidative phosphorylation events.

### 3.7 Stress Combined With Sustained Tumour Growth Upregulates a Large Cluster of Proteins in the Hippocampus Associated With Oxidative Phosphorylation

**FIGURE 8:**
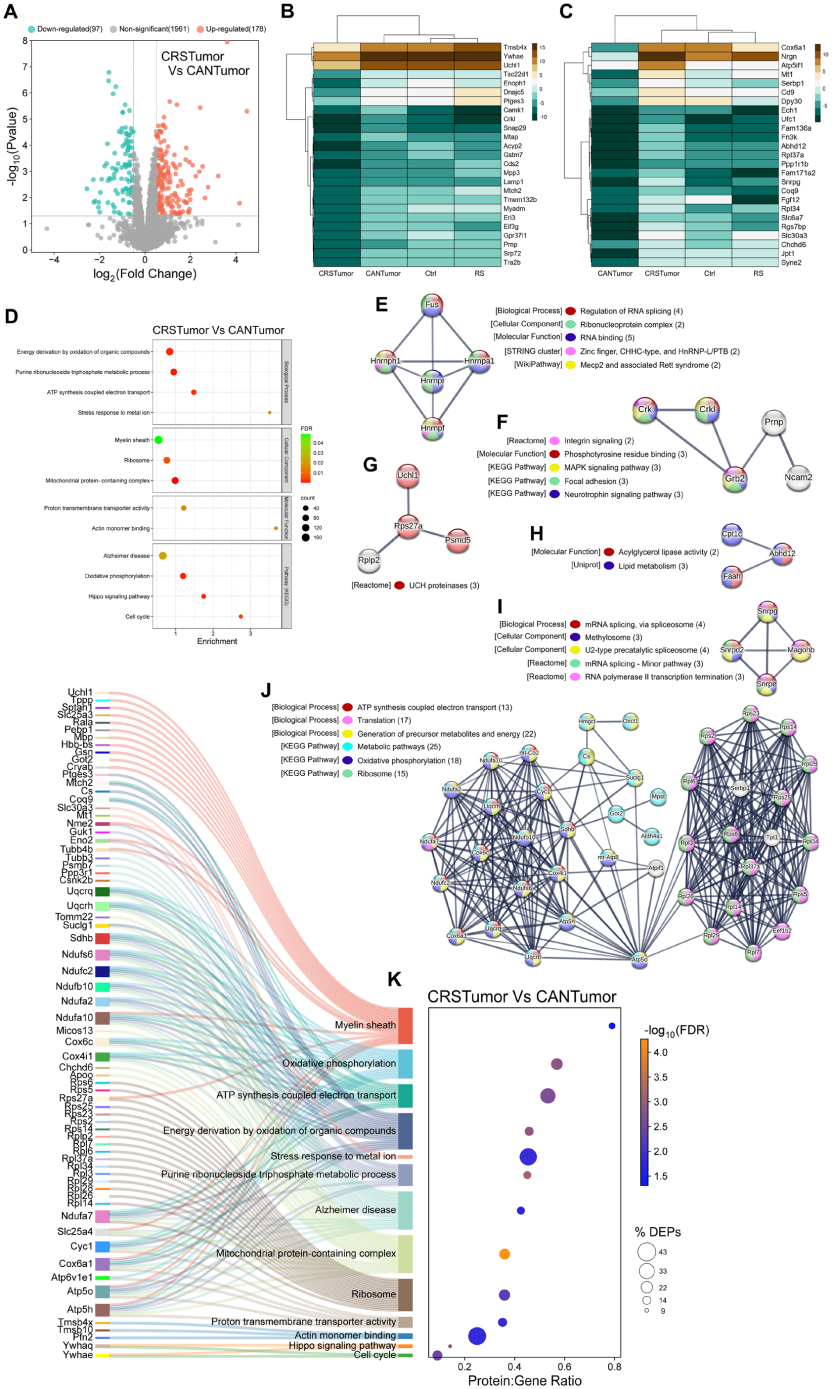
Daily restraint stress, in combination with sustained tumour burden, leads to changes in ribosomal, mitochondrial and ATP synthesis‐related proteins which may impact oxidative phosphorylation pathways in the hippocampus. (A) Volcano plot depicting the up‐ and down‐regulated proteins in the CRSTumor protein samples compared to the CANTumor group. A total of 97 proteins (green dots) were downregulated and 178 up‐regulated (orange dots). Proteins expressing a log_2_ fold change > ±0.5 and a *p*‐value < 0.05, using the Benjamini‐Hochberg false discovery rate (FDR) correction, were considered significant. (B) Clustered heatmap of the top 25 significantly downregulated proteins in the CRSTumor group compared to CANTumor samples. The relative expression of each protein in the RS and Ctrl samples is also shown. (C) Clustered heatmap of the top 25 significantly up‐regulated proteins in the CRSTumor group compared to CANTumor samples. The Ward.D clustering method was used which utilises the sum of squared distances to minimise the merging of clusters. (D) Gene ontology (GO) enrichment analysis was performed in STRING v.12.0 using a ranked list of all proteins detected in the CRSTumor and CANTumor groups of mice. Proteins upregulated in the CRSTumor hippocampus ranked at the top of the list and downregulated proteins ranked at the bottom. Significantly enriched biological processes (BPs), cellular components (CCs), molecular functions (MFs) and KEGG (Kyoto Encyclopaedia of Genes and Genomes) pathways are displayed against the enrichment score, along with the number of proteins (count) detected in our dataset which are linked to each GO term. The colour of each dot corresponds to its FDR value. (E–G) Clusters of functionally‐related proteins that were significantly downregulated in the hippocampus of CRSTumor mice compared to the CANTumor samples. The colours of each protein represent their functions in terms of the BPs, CCs, MFs, KEGG pathways, STRING clusters or Reactome pathways that they are associated with. (H–J) Clusters of functionally‐related proteins that were significantly upregulated in the hippocampus of CRSTumor versus CANTumor mice. (K) Differentially expressed proteins (DEPs) associated with enriched GO terms are listed to the left of the Sankey plot and connected to one or more BP, CC, MF or KEGG pathway to the right. The Protein:Gene Ratio (*X*‐axis) represents the proportion of proteins associated with that particular GO term which were detected in our dataset. The bubble size depicts the percentage of detected proteins linked to each GO term that showed differential expression, that is, (no. of DEPs/no. of detected proteins associated with that GO term) × 100. The bubble colour represents −log_10_ (FDR) value. Ctrl, control; RS, restraint stress; CANTumor, mice with sustained tumour growth; CRSTumor, stressed mice with sustained tumour growth.

To investigate the impact of a psychological stressor (physical restraint) combined with the physiological stress of tumour growth on protein expression in the hippocampus, a volcano plot was generated to visualise all proteins up‐ and down‐regulated in the CRSTumor versus CANTumor group. A total of 97 proteins were downregulated in the CRSTumor group, whereas 178 proteins were upregulated (Figure 8A). The top 25 downregulated (Figure 8B) and top 25 up‐regulated (Figure 8C) proteins are represented by clustered heatmaps to also show their relative expression in the RS and Ctrl groups. Next, gene ontology (GO) enrichment analysis was performed using STRING v.12.0, in which a ranked list of all proteins detected between the CRSTumor and CANTumor groups (2236) were ordered such that the upregulated proteins ranked at the top and downregulated proteins at the bottom of the list. Significantly enriched (FDR < 0.05) biological processes (BP), cellular components (CC), molecular functions (MF), and KEGG pathways were then ordered based on their enrichment scores (Figure 8D). Proteins known to be involved in (1) ATP synthesis and cellular energy production, (2) cellular stress responses to metal ions, (3) myelin sheath integrity, (4) actin monomer binding and (5) transport of ions across the membrane were differentially expressed in the hippocampus of CRSTumor versus CANTumor mice. KEGG pathway analysis suggested that proteins involved in (1) oxidative phosphorylation, (2) regulating the cell cycle and (3) the Hippo signalling pathway are also impacted in the hippocampus of tumour‐bearing mice exposed to chronic daily restraint stress (Figure 8D). STRING v.12.0 was also used to identify functionally‐related clusters of DEPs. Upregulated and downregulated sets of proteins were inputted separately into STRING, and functional enrichment analysis was performed against the full background of 2300 identified proteins in our mass spectrometry dataset. There were three interesting clusters of functionally‐related proteins downregulated in CRSTumor versus CANTumor mice. The first was a cluster of 5 RNA‐binding proteins involved in the regulation of RNA splicing (Figure 8E). The second was a set of 5 proteins that may be important in the regulation of synaptic plasticity via MAPK signalling and integrin signalling at focal adhesion sites (Figure 8F). The third down‐regulated cluster comprised 4 proteins that may be involved in the regulation of the ubiquitin proteasome complex in the cell (Figure 8G). From the 178 up‐regulated proteins in the CRSTumor versus CANTumor comparison, there were also 3 interesting clusters of functionally‐related proteins. The smallest cluster of 3 proteins (Cpt1c, Faah and Abhd12) are involved in acylglycerol lipase activity and lipid metabolism (Figure 8H). A second cluster consisted of 3 small nuclear ribonucleoproteins (Snrp) and Magohb, which may be involved in mRNA splicing events (Figure 8I). The largest cluster was composed of 44 DEPs and included ribosomal proteins involved in mRNA translation, as well as mitochondrial proteins linked to metabolic pathways, ATP‐synthesis, and oxidative phosphorylation (Figure 8J). Next, a Sankey plot was generated to display the protein: gene ratio for each of the GO terms identified by functional enrichment analysis (Figure 8K). Listed on the left‐hand side of the Sankey plot are all the DEPs associated with each of the functionally enriched GO terms.

### 3.8 Spontaneous Tumour Regression Impacts the Hippocampal Proteome in a Significantly Different Way to Py230 Cancer Cell Rejection

**FIGURE 9:**
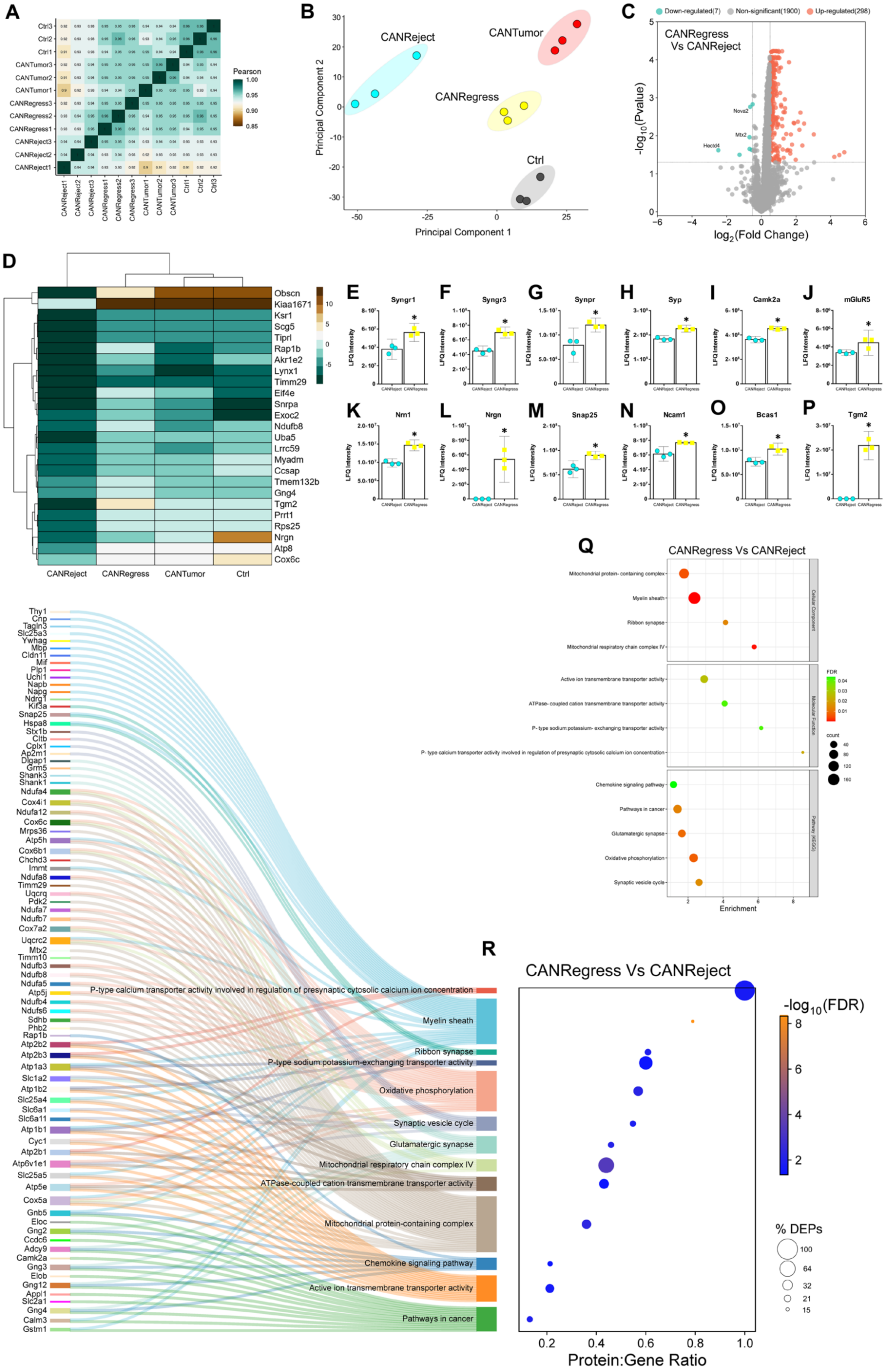
The hippocampal proteome of mice in which mammary tumours spontaneously regressed is distinctly different to that from mice in which Py230 cells were rejected. (A) Correlation matrix displaying the Pearson *r* values for each protein sample pairwise comparison between the Ctrl, CANTumor, CANRegress, and CANReject groups of mice. Shades of green represent higher Pearson correlation scores whereas shades of brown depict less correlated comparisons. (B) Principal component analysis (PCA) of the key features in the mass spectrometry data sets that best describe the variance between Ctrl, CANTumor, CANRegress, and CANReject hippocampal proteomes. (C) Volcano plot depicting the up‐ and down‐regulated proteins in the CANRegress protein samples compared to the CANReject group. Only 7 proteins (green dots) were downregulated whereas 298 were upregulated (orange dots). Proteins expressing a log_2_ fold change > ±0.5 and a *p*‐value < 0.05, using the Benjamini‐Hochberg false discovery rate (FDR) correction, were considered significant. (D) Clustered heatmap of the top 25 significantly upregulated proteins in the CANRegress group compared to CANReject hippocampal samples. The relative expression levels of each protein in the Ctrl and CANTumor samples are also shown. The Ward.D clustering method was used which utilises the sum of squared distances to minimise the merging of clusters. (E–P) A selection of functionally interesting upregulated proteins in the hippocampus of the CANRegress group compared to CANReject samples. Displayed on the *Y*‐axes of the bar charts are the raw label‐free quantitation (LFQ) intensity values for each protein. Data represent the means ± 95% confidence intervals. (Q) Gene ontology (GO) enrichment analysis was performed in STRING v.12.0 using a ranked list of all proteins detected in the CANRegress and CANReject groups of mice. Proteins upregulated in the CANRegress hippocampus ranked at the top of the list and downregulated proteins ranked at the bottom. Significantly enriched cellular components (CCs), molecular functions (MFs) and KEGG (Kyoto Encyclopaedia of Genes and Genomes) pathways are displayed against the enrichment score, along with the number of proteins (count) detected in our dataset which are linked to each GO term. The colour of each dot corresponds to its FDR value. (R) Differentially expressed proteins (DEPs) associated with enriched GO terms are listed to the left of the Sankey plot and connected to one or more CC, MF or KEGG pathway to the right. The Protein:Gene Ratio (X‐axis) represents the proportion of proteins associated with that particular GO term which were detected in our dataset. The bubble size depicts the percentage of detected proteins linked to each GO term that showed differential expression, that is, (no. of DEPs/no. of detected proteins associated with that GO term) × 100. The bubble colour represents −log_10_(FDR) value. Ctrl, control; CANTumor, mice with sustained tumour growth; CANRegress, mice in which tumours regressed; CANReject, mice that rejected cancer cells.

The next aim was to investigate the similarities between the hippocampal proteomes of mice that initially developed mammary tumours which spontaneously regressed (CANRegress) versus mice that received the same Py230 cancer cell inoculation but failed to develop palpable tumours (CANReject). A correlation matrix was generated to visualise the extent to which the protein sample replicates were correlated (Figure 9A). Within group protein sample replicates were highly correlated (Pearson *r* ≥ 0.94). CANReject sample replicates were the least correlated to other groups. This can also be visualised using principal component analysis (PCA) which describes how the CANReject samples (in blue) are further separated along the PC1 axis from the other three groups of mice (Figure 9B). Next, a volcano plot was generated to analyse the proteins that were significantly up‐ or down‐regulated in the CANRegress group compared to the CANReject mice. Seven proteins were found to be downregulated in the hippocampus of CANRegress versus CANReject mice, whereas 297 proteins were upregulated (Figure 9C). A clustered heatmap displaying the top 25 up‐regulated proteins in the CANRegress vs. CANReject groups also illustrates the relative abundance of each protein in the CANTumor mice and in Ctrl animals (Figure 9D). Among the 297 up‐regulated proteins in the hippocampus of mice that experienced spontaneous mammary tumour regression (compared to CANReject mice) were neuronal and synaptic plasticity‐associated proteins, including synaptogyrin‐1 (Syngr1), synaptogyrin‐3 (Syngr3), synaptoporin (Synpr), synaptophysin (Syp), neuritin‐like protein (Nrn1), neurogranin (Nrgn), synaptosomal‐associated protein 25 (Snap25) and neural cell adhesion molecule (Ncam1). Moreover, the calcium/calmodulin‐dependent protein kinase, Camk2a and the metabotropic glutamate receptor, mGluR5, were upregulated in the hippocampus of mice that displayed spontaneous tumour regression. Finally, brain‐enriched myelin‐associated protein 1 (Bcas1) and the calcium‐regulated amino acid crosslinking enzyme, transglutaminase‐2 (Tgm2), were elevated in the hippocampus of CANRegress versus CANReject mice (Figure 9E–P). Next, GO enrichment analysis was performed using STRING v.12.0 in which a ranked list of all proteins detected between the CANRegress and CANReject groups (2205) were ordered such that the upregulated proteins ranked at the top and downregulated proteins at the bottom of the list. Significantly enriched (FDR < 0.05) cellular components (CC), molecular functions (MF), and KEGG pathways were then ordered based on their enrichment scores (Figure 9Q). Proteins known to be involved in (1) the mitochondrial respiratory chain complex, (2) ribbon synapses, (3) myelin sheath integrity, (4) regulation of cytosolic calcium concentration and (5) active transport of ions across the membrane, were differentially expressed in the hippocampus of CANRegress versus CANReject mice. Moreover, KEGG pathway analysis suggested that proteins involved in (1) chemokine signalling, (2) oxidative phosphorylation, (3) cancer‐related pathways and (4) synaptic vesicle turnover at glutamatergic synapses, are also impacted in the hippocampus of mice in which mammary tumours spontaneously regressed after 7–9 weeks of tumour growth (Figure 9Q). A Sankey plot was generated to display the protein: gene ratio for each of the GO terms identified by the above functional enrichment analysis. Listed on the left‐hand side of the Sankey plot are all of the DEPs associated with each of the enriched GO terms (Figure 9R).

**FIGURE 10:**
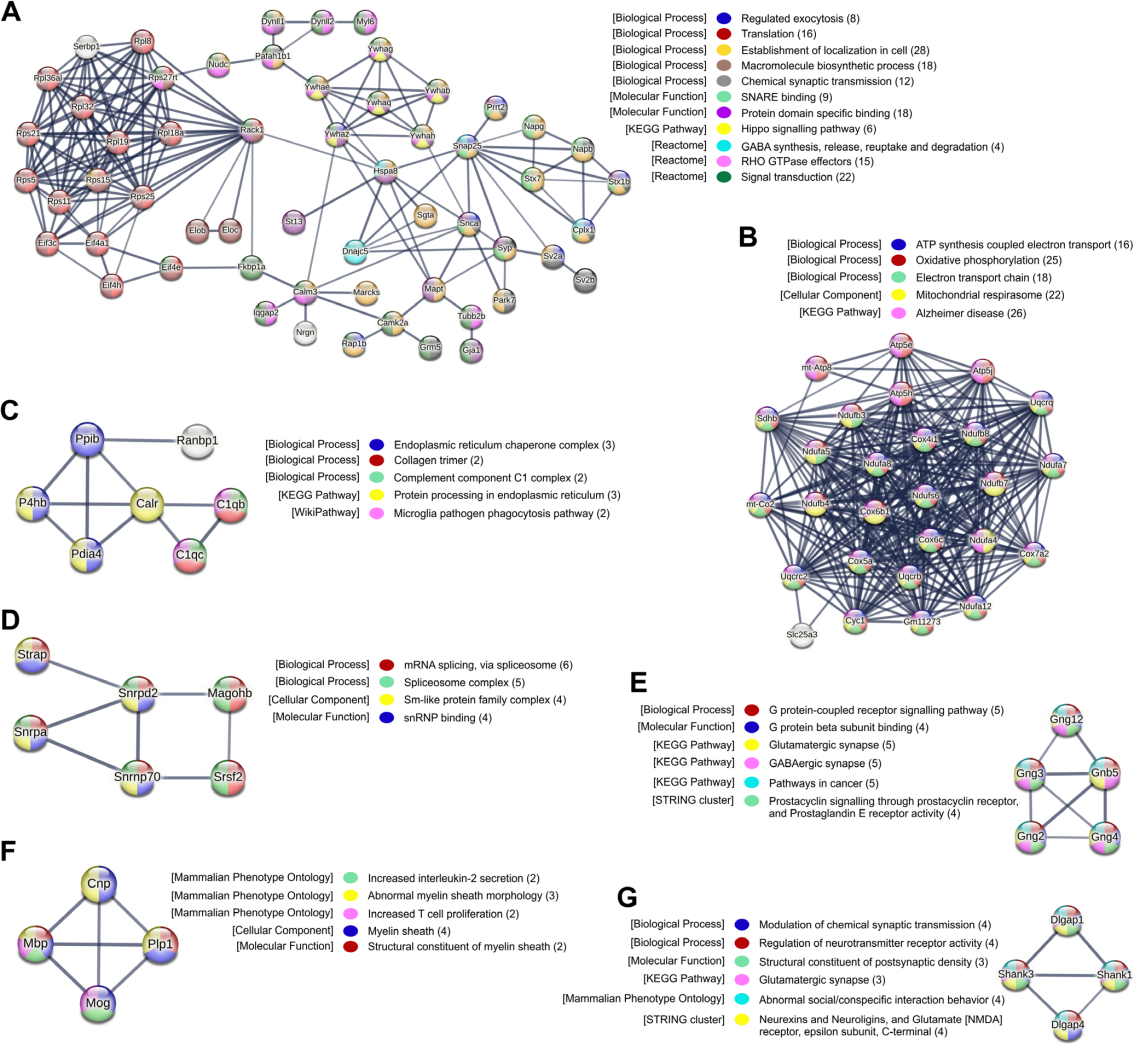
Functionally‐related protein clusters upregulated in the hippocampus of CANRegress versus CANReject mice. STRING v.12.0 was used to identify clusters of proteins within the 297 significantly upregulated proteins in the CANRegress versus CANReject mice. (A) Cluster of 57 proteins involved in common biological functions including intracellular signalling, mRNA translation, and neurotransmitter synthesis, exocytosis, reuptake and degradation. (B) Cluster of 27 proteins involved in common biological functions including the electron transport chain, ATP synthesis, and oxidative phosphorylation. (C) Cluster of 7 proteins involved in common biological functions including protein folding, the complement cascade, and microglial phagocytosis of pathogens. (D) Cluster of 6 proteins involved in common biological functions including pre‐mRNA splicing via the spliceosome complex. (E) Cluster of 5 proteins involved in common biological functions including G protein‐coupled signalling at glutamate and GABA synapses. (F) Cluster of 5 proteins involved in common biological functions including structural integrity of the myelin sheath and increased T cell proliferation. (G) Cluster of 4 proteins involved in common biological functions including the regulation of chemical synaptic transmission at glutamate synapses. CANRegress, mice in which tumours regressed; CANReject, mice that rejected cancer cells.

STRING v.12.0 was also used to identify functionally‐related clusters of DEPs. There were no clusters identified in the 7 down‐regulated proteins. However, when the 297 up‐regulated proteins were inputted into STRING and functional enrichment analysis was performed against the full background of 2300 identified proteins in our mass spectrometry dataset, 7 clusters containing four or more proteins were identified in the hippocampus of CANRegress versus CANReject mice. The largest cluster contained 57 proteins composed of (1) ribosomal proteins involved in translation, (2) signaling molecules such as kinases and RhoGTPases and (3) synaptic proteins that regulate exocytosis (Figure 10A). The second largest cluster of upregulated proteins was composed of 27 proteins involved in mitochondrial respiration, ATP synthesis, and oxidative phosphorylation (Figure 10B). A third cluster of up‐regulated proteins in the CANRegress versus CANReject hippocampus were 7 proteins with links to protein processing at the endoplasmic reticulum, as well as to the complement component C1 complex (Figure 10C). A fourth cluster of 6 up‐regulated proteins are associated with mRNA splicing via the spliceosome complex (Figure 10D). A cluster of 5 proteins found at glutamatergic and GABAergic synapses involved in G protein signaling was also upregulated (Figure 10E). Interestingly, four proteins (Cnp, Plp1, Mbp and Mog) known to be important in regulating the structural integrity of the myelin sheath were upregulated in the hippocampus of CANRegress versus CANReject mice (Figure 10F). Finally, 4 proteins involved in neurotransmitter receptor activity and chemical synaptic transmission at glutamatergic synapses (Shank1, Shank3, Dlgap1 and Dlga4) were also upregulated in the hippocampus of CANRegress versus CANReject mice (Figure 10G).

### 3.9 The Hippocampal Proteomes of ‘Tumour’ and ‘Tumour Regressed’ Mice Are More Similar to One Another Than to Mice That Rejected Cancer Cell Inoculation

**FIGURE 11:**
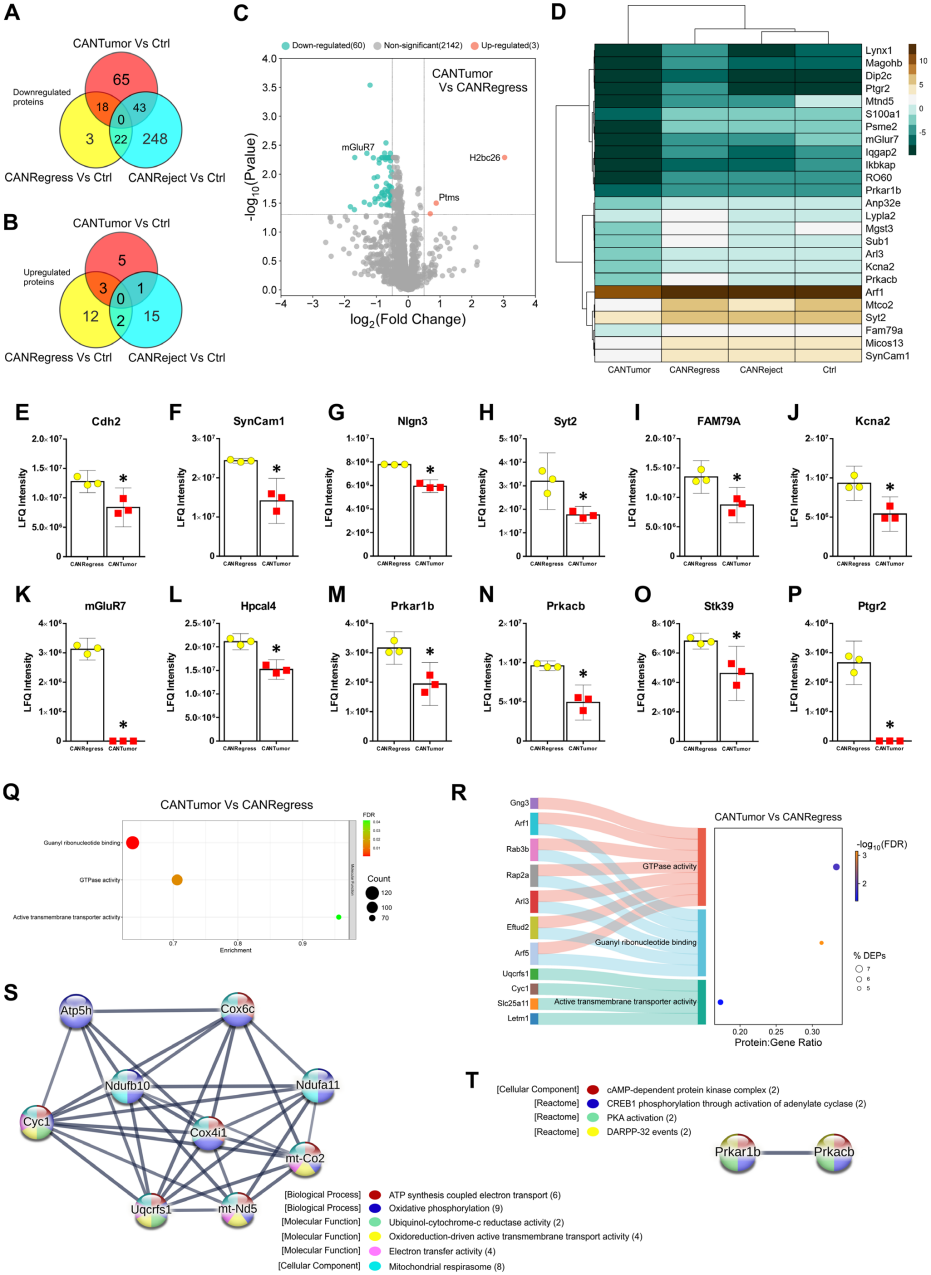
The hippocampal proteome of CANTumor mice is more similar to the proteome of CANRegress mice than to that of CANReject mice. (A, B) Venn diagram detailing the number of downregulated and upregulated proteins in common between the CANTumor versus Ctrl, the CANRegress versus Ctrl, and the CANReject versus Ctrl comparisons. (C) Volcano plot depicting the up‐ and down‐regulated proteins in the CANTumor protein samples compared to the CANRegress group. Only 3 proteins (orange dots) were up‐regulated whereas 60 were down‐regulated (green dots). Proteins expressing a log_2_ fold change > ±0.5 and a *p*‐value < 0.05, using the Benjamini‐Hochberg false discovery rate (FDR) correction, were considered significant. (D) Clustered heatmap of the top 25 significantly downregulated proteins in the CANTumor group compared to CANRegress samples. The relative expression of each protein in the Ctrl and CANReject samples is also shown. The Ward.D clustering method was used which utilises the sum of squared distances to minimise the merging of clusters. (E–P) A selection of functionally interesting downregulated proteins in the hippocampus of the CANTumor group compared to CANRegress samples. Displayed on the *Y*‐axes of the bar charts are the raw label‐free quantitation (LFQ) intensity values for each protein. Data represent the means ± 95% confidence intervals. (Q) Gene ontology (GO) enrichment analysis was performed in STRING v.12.0 using a ranked list of all proteins detected in the CANTumor and CANRegress groups of mice. Proteins upregulated in the CANTumor hippocampus ranked at the top of the list and downregulated proteins ranked at the bottom. There were no significantly enriched biological processes, cellular components, or KEGG pathways. Enriched molecular functions (MFs) are displayed against the enrichment score, along with the number of proteins (count) detected in our dataset which are linked to each GO term. The colour of each dot corresponds to its false discovery rate (FDR) value. (R) Differentially expressed proteins (DEPs) associated with the enriched GO terms are listed to the left of the Sankey plot and connected to one or more MF to the right. The Protein:Gene Ratio (*X*‐axis) represents the proportion of proteins associated with that particular GO term which were detected in our dataset. The bubble size depicts the percentage of detected proteins linked to each GO term that showed differential expression, that is, (no. of DEPs/no. of detected proteins associated with that GO term) × 100. The bubble colour represents −log_10_(FDR) value. (S) Cluster of 9 proteins that were downregulated in the hippocampus of CANTumor mice compared to CANRegress mice. This group of proteins are involved in common biological functions including, ATP synthesis, mitochondrial respiration, oxidative phosphorylation, and transmembrane transport. (T) Two cAMP‐dependent protein kinase regulatory subunits were downregulated in the hippocampus of CANTumor mice compared to CANRegress mice. These proteins are involved in common biological functions including, CREB phosphorylation and DARPP‐32 activity. Ctrl, control; CANTumor, mice with sustained tumour growth; CANRegress, mice in which tumours regressed; CANReject, mice that rejected cancer cells.

Analysis of hippocampal protein expression in the CANTumor, CANRegress and CANReject groups of mice suggested that the CANTumor and CANRegress brain samples are more similar to each other than to the CANReject group (Figure 9B). Moreover, when each group was compared separately to Ctrl mice, the CANReject group displayed 308 DEPs (291 down‐regulated and 17 up‐regulated proteins), whereas the CANTumor group displayed 116 DEPs (108 down‐regulated and 8 up‐regulated proteins), and the CANRegress group exhibited 40 DEPs (25 down‐regulated and 15 up‐regulated proteins) (Figure 11A,B). This suggests that mice that rejected Py230 cell inoculation also exhibited many changes in hippocampal protein expression when compared to controls. This may be due to a robust and effective immunogenic cell death (ICD) response triggered by the initial tumour cell inoculation in 9 out of the 48 immunocompetent mice (Ahmed and Tait 2020; Kroemer et al. 2022). Interestingly, 18 out of the 25 downregulated proteins in the CANRegress versus Ctrl comparison were also downregulated in the CANTumor versus Ctrl comparison (Figure 11A). To understand just how similar these hippocampal proteomes are, and whether mammary tumour development and spontaneous tumour regression impact this brain structure in a similar way, changes in protein expression in the CANTumor versus CANRegress groups were next investigated. A volcano plot was generated to analyse the proteins that were significantly up‐ or down‐regulated in the CANTumor group compared to the CANRegress mice. Three proteins were found to be upregulated in the hippocampus of CANTumor versus CANRegress mice, whereas 60 proteins were downregulated (Figure 11C). A clustered heatmap displaying the top 25 down‐regulated proteins in the CANTumor versus CANRegress groups also illustrates the relative abundance of each protein in the CANReject mice and in Ctrl animals (Figure 11D). Among the 60 down‐regulated proteins in mice that developed mammary tumours (compared to CANRegress mice) were neural cell adhesion molecules and synaptic plasticity‐associated proteins, including cadherin‐2 (Cdh2), cell adhesion molecule 1 (SynCam1), neuroligin‐3 (Nlgn3), synaptotagmin‐2 (Syt2), FAM79A (Mover) and the voltage‐gated potassium channel (Kcna2) (Figure 11E–J). Moreover, the metabotropic glutamate receptor, mGluR7, as well as calcium‐regulated and cAMP‐dependent signalling molecules were downregulated, including hippocalcin‐like protein 4 (Hpcal4), cAMP‐dependent protein kinase type I‐beta regulatory subunit (Prkar1b), cAMP‐dependent protein kinase catalytic subunit beta (Prkacb) and STE20/SPS1‐related proline‐alanine‐rich protein kinase (Stk39) (Figure 11K–O). Finally, the cellular stress associated protein, prostaglandin reductase 2 (Ptgr2), was completely ablated in the hippocampus of CANTumor versus CANRegress mice (Figure 11P), suggesting elevated levels of reactive oxygen species and cell death in the mice that experienced sustained tumour burden.

Next, GO enrichment analysis was performed using STRING v.12.0 in which a ranked list of all proteins detected between the CANTumor and CANRegress groups (2205) were ordered such that the upregulated proteins ranked at the top and downregulated proteins at the bottom of the list. No significantly enriched (FDR < 0.05) biological processes, cellular components (CC), or KEGG pathways were found. Enriched molecular functions from the GO analysis included: (1) guanyl ribonucleotide binding, (2) GTPase activity and (3) active transmembrane transporter activity. A Sankey plot was generated to display the protein: gene ratio for each of the GO terms identified by the above functional enrichment analysis. Listed on the left‐hand side of the Sankey plot are all of the DEPs associated with each of the enriched GO terms (Figure 11R). STRING v.12.0 was also used to identify functionally‐related clusters of DEPs. There were no clusters identified in the 3 up‐regulated proteins. When functional enrichment analysis of the 60 downregulated proteins was performed against the full background of 2300 identified proteins in our mass spectrometry dataset, two clusters were identified in the hippocampus of CANTumor versus CANRegress mice. The largest cluster contained 9 proteins involved in mitochondrial respiration, ATP synthesis, and oxidative phosphorylation (Figure 11S). The second cluster contained just two protein kinases, Prkar1b and Prkacb, involved in signalling events such as cAMP‐dependent protein kinase A (PKA) activation and adenylate cyclase mediated CREB1 phosphorylation (Figure 11T).

**FIGURE 12:**
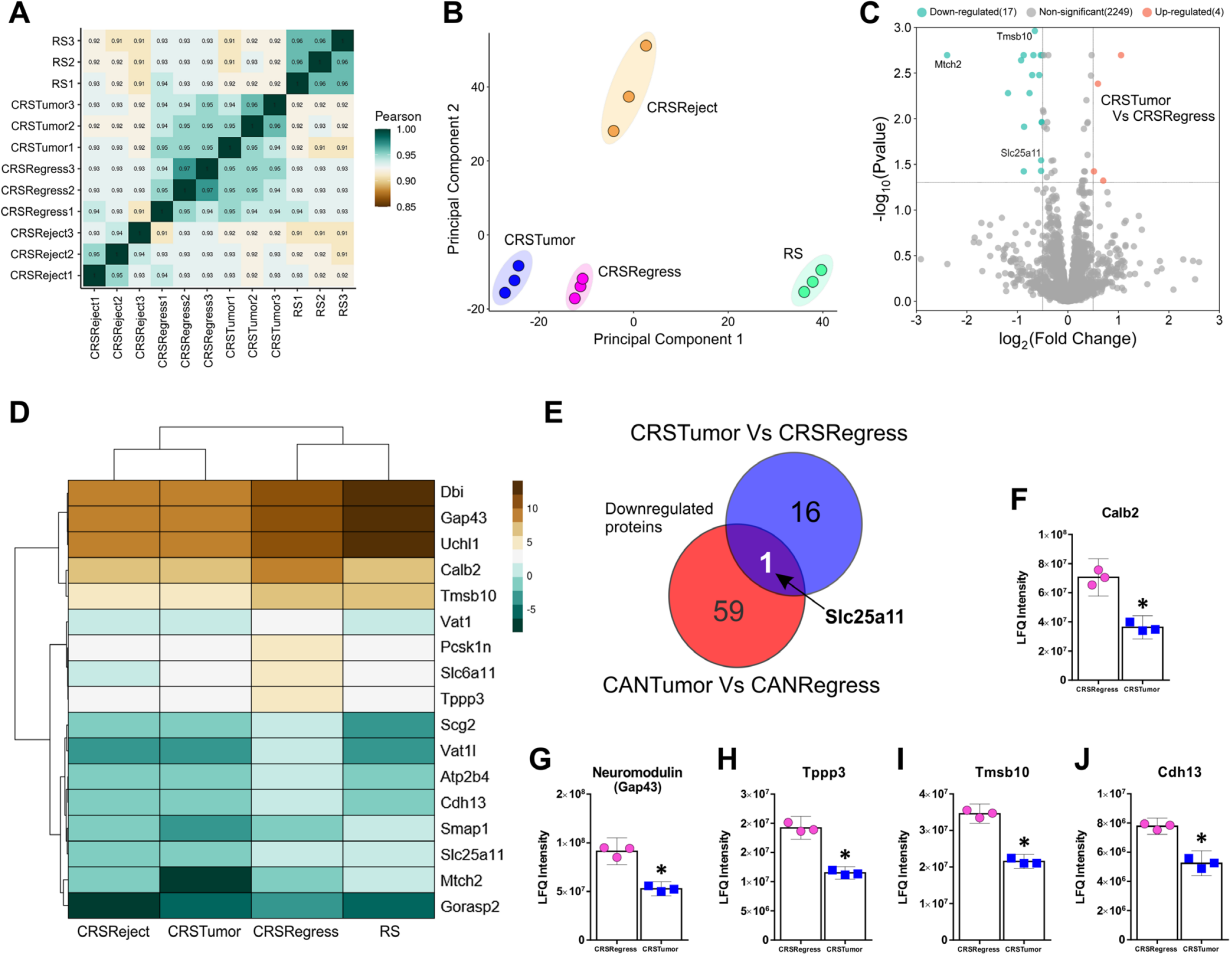
The hippocampal proteome of mice that developed tumours and received daily stress is similar to the hippocampal proteome of stressed mice in which mammary tumours spontaneously regressed. (A) Correlation matrix displaying the Pearson *r* values for each protein sample pairwise comparison between RS, CRSTumor, CRSRegress and CRSReject groups of mice. Shades of green represent higher Pearson correlations whereas shades of brown depict less correlated comparisons. (B) Principal component analysis (PCA) of the key features in the mass spectrometry data sets that best describe the variance between RS, CRSTumor, CRSRegress, and CRSReject hippocampal proteomes. (C) Volcano plot depicting the up‐ and down‐regulated proteins in the CRSTumor protein samples compared to the CRSRegress group. Only 4 proteins (orange dots) were upregulated whereas 17 were downregulated (green dots). Proteins expressing a log_2_ fold change > ±0.5 and a *p*‐value < 0.05, using the Benjamini‐Hochberg false discovery rate (FDR) correction, were considered significant. (D) Clustered heatmap of the 17 significantly downregulated proteins in the CRSTumor group compared to CRSRegress samples. The relative expression of each protein in the RS and CRSReject samples is also shown. The Ward.D clustering method was used which utilises the sum of squared distances to minimise the merging of clusters. (E) Venn diagram detailing the number of downregulated proteins in common between the CRSTumor versus CRSRegress and the CANTumor versus CANRegress comparisons. There was only one protein commonly downregulated, that is, mitochondrial 2‐oxoglutarate/malate carrier protein (Slc25a11). (F–J) A selection of functionally interesting downregulated proteins in the hippocampus of the CRSTumor group compared to CRSRegress samples. Displayed on the *Y*‐axes of the bar charts are the raw label‐free quantitation (LFQ) intensity values for each protein. Data represent the means ± 95% confidence intervals. RS, restraint stress; CRSTumor, stressed mice with sustained tumour growth; CRSRegress, stressed mice in which tumours regressed; CRSReject, stressed mice that rejected cancer cells.

Interestingly, results were similar in the stressed groups of mice that were inoculated with Py230 cancer cells, that is, the CRSTumor and CRSRegress groups were more similar to one another than to the CRSReject group (Figure 12A,B). Comparing the CRSTumor versus CRSRegress mice in more detail revealed that there were 4 proteins upregulated and 17 proteins downregulated in the CRSTumor group (Figure 12C). To visualise the relative expression of these 17 down‐regulated proteins in the RS and CRSReject groups of mice, a clustered heatmap was generated (Figure 12D). Next, to investigate whether the same proteins were down‐regulated in CRSTumor versus CRSRegress mice and in CANTumor versus CANRegress mice, a Venn diagram was generated to visualise the commonly down‐regulated proteins between both comparisons (Figure 12E). Interestingly, there was only one protein in common, that is, mitochondrial 2‐oxoglutarate/malate carrier protein (Slc25a11). Among the 17 downregulated proteins in stressed mice that developed mammary tumours (compared to CRSRegress mice) were proteins involved in cytoskeletal dynamics in cellular processes such as axon guidance, neurite extension, cellular proliferation, and cell fate decisions. Notably, the proteins calretinin (Calb2), neuromodulin (GAP43), tubulin polymerisation‐promoting protein (Tppp3), thymosin beta‐10 (Tmsb10) and cadherin‐13 (Cdh13) were significantly downregulated in CRSTumor mice (Figure 12F–J). GO enrichment analysis was performed using STRING v.12.0 in which a ranked list of all proteins detected between the CRSTumor and CRSRegress groups (2270) were ordered such that the upregulated proteins ranked at the top and downregulated proteins at the bottom of the list. No significantly enriched biological processes, cellular components (CC), molecular functions, or KEGG pathways were found. Similarly, there were no clusters of functionally‐related proteins from the list of up‐ and down‐regulated proteins in the hippocampus of CRSTumor versus CRSRegress mice.

It should also be noted that the CRSReject mice displayed a large number of DEPs in the hippocampus compared to the restraint stress (RS) mice (47 up‐ and 334 down‐regulated), and similarly, the CANReject mice presented with a large number of DEPs compared to the control (Ctrl) mice (18 up‐ and 291 down‐regulated). Moreover, the mice in which tumours initially grew and then spontaneously regressed after 7–9 weeks post‐inoculation displayed more similar results to the CANTumor and CRSTumor groups than to the CANReject and CRSReject groups, respectively. These observations advocate two main working hypotheses going forward: (1) that developing a non‐CNS tumour that spontaneously regresses after a period of sustained growth (Radha and Lopus 2021) may impact the brain in a similar way to tumours that continue to grow over time; and (2) organs outside of the central nervous system that harbour cells which have mutated into cancer cells, and which are subsequently attacked and destroyed by our immune system, may still trigger long‐lasting pathophysiological changes in our body (Matzinger 2002) that indirectly impact protein expression in the brain, such as the hippocampal region of the limbic system.

## 4 Discussion

The primary aim of this study was to model the individual and combined effects of breast cancer and psychological stress on the hippocampal proteome; a region of the brain important for learning and memory formation (Jarrard 1995, 1993). The rationale for combining a syngeneic non‐metastatic breast tumour mouse model with and without an inescapable daily restraint stress paradigm was to attempt to simulate the psychological stress that humans experience as a result of being diagnosed with cancer (Atrooz et al. 2021). Therefore, by elucidating global changes in protein expression in this key stress‐responsive brain region as a result of non‐CNS tumour growth and/or chronic daily restraint, we hope to shed new light on the aetiology and molecular underpinnings of cancer‐related cognitive impairment (CRCI) (Országhová et al. 2021). Importantly, the results presented here describe the impact of pre‐treatment tumour growth on the brain, as opposed to chemotherapy‐induced cognitive impairment (Mampay et al. 2021). Although tumours grow more readily in immunocompromised mice, it was important to examine the effects of mammary tumour growth on hippocampal protein expression in an immunocompetent mouse model (i.e., C57Bl/6), since there is evidence that the immune system is important for maintaining cognitive functioning in rodents (Brynskikh et al. 2008; Derecki et al. 2010; Ziv et al. 2006). This is also true in humans since people with immune deficiencies, such as the elderly, people living with HIV, or individuals with primary immunodeficiency disorders (Sowers et al. 2020) often display deficits in learning and memory formation, sometimes referred to as ‘brain fog’ (Fleming et al. 2023; Nightingale et al. 2023; Shafqat et al. 2023). While the brain is considered an immune‐privileged organ due to the extra layer of protection afforded by the blood–brain barrier (BBB); it is now well established that there is significant crosstalk between the immune system and the central nervous system (CNS) (Carson et al. 2006). However, the mechanistic link between non‐CNS tumour growth and cognitive impairment has not yet been elucidated. We hypothesise that CRCI may begin with dysregulated crosstalk between tumour‐associated immune cells and the neurovascular units of key brain regions (Arvanitis et al. 2020) which, over time, leads to neuroinflammation and maladaptive changes in synaptic plasticity (Schetters et al. 2018).

### 4.1 Establishing a Model to Study the Impact of Breast Cancer on the Brain

For the Py230 cell‐based model of triple‐negative breast cancer, we estimated a 50%–80% tumour take rate, meaning that we initially expected around 20%–50% of the young immunocompetent female C57Bl/6 mice would not form mammary tumours. What we observed was that 39 out of 48 (~81%) mice developed mammary tumours by Week 7 post‐inoculation (Figure 1C,D). However, 26 out of the 39 (~67%) tumours spontaneously regressed by Week 9 (Figure 1D,F), before the start of the restraint stress paradigm. This provided us with an interesting dataset consisting of the brains of immunocompetent mice that developed tumours (CANTumor ~27%), mice that did not develop tumours (CANReject ~19%), and mice that developed tumours which spontaneously regressed after 7–9 weeks (CANRegress ~54%). These mice were then split into cancer groups which either received a 2‐h daily session of inescapable restraint stress for 3 weeks (CRS) or remained unstressed (CAN). We found that 3 weeks of restraint stress caused both a decrease in body weight and an increase in tumour volume in the CRSTumor group (Figure 1I,J). Mice that developed tumours also showed an increase in circulating stress hormones as evidenced by an increase in urinary corticosterone concentrations (Figure 2A,B). However, the CRSTumor mice displayed the highest levels of urinary corticosterone (Figure 2C), suggesting that restraint stress caused an additive effect on circulating stress hormone concentrations. This result may bear clinical relevance since elevated circulating cortisol concentrations have been reported in women diagnosed with breast cancer but who are yet to receive anti‐neoplastic treatment (Mészáros Crow et al. 2023; Olson and Marks 2019; Ramírez‐Expósito et al. 2021).

Since both cancer growth and chronic stress can impact immune functioning in different ways (Barrett et al. 2021; Burkholder et al. 2014), we next calculated the ratio of CD4^+^ to CD8^+^ T lymphocytes extracted from the spleens of each of the eight groups of mice (Figure 4C). The interpretation of this data must be treated with caution since the roles of CD4^+^ and CD8^+^ T cells in anti‐tumour immunity are not well defined. Moreover, there are several subsets of CD4^+^ and CD8^+^ T cells that possess different functions (Notarbartolo and Abrignani 2022). In general, CD8^+^ T cells are considered cytotoxic toward tumour cells and act by infiltrating and recognising tumour antigens bound to MHC class I molecules on tumour cells (Raskov et al. 2021). In contrast, CD4^+^ T cells recognise tumour antigens bound to MHC class II molecules on antigen presenting cells and help to initiate tumour‐specific cytotoxic T lymphocyte (CTL) responses (Tay et al. 2021). Therefore, tumour elimination usually requires the coordinated actions of both CD4^+^ and CD8^+^ T cells (Schietinger et al. 2010). The clinical significance of changes in the ratio of CD4^+^ to CD8^+^ T lymphocytes is still a matter of debate, however (Overgaard et al. 2015). Studies have shown that an increase in the CD4^+^/CD8^+^ ratio correlated with tumour progression and decreased survival in patients with breast cancer (Yang et al. 2017). This supports our data which shows that the CD4^+^/CD8^+^ ratio is elevated to 5.2 ± 0.6 in non‐stressed tumour‐bearing mice (CANTumor) compared to 2.6 ± 0.5 in mice in which tumours spontaneously regressed (CANRegress). However, tumour‐bearing mice that received 3 weeks of chronic restraint stress (CRSTumor) displayed an average CD4^+^/CD8^+^ ratio of 2.4 ± 0.2, which was similar to control animals (1.8 ± 0.1) and to stressed mice in which tumours spontaneously regressed (2.1 ± 0.5; CRSRegress) (Figure 4C). Moreover, chronically stressed tumour‐bearing mice displayed the largest tumour volumes (Figure 1J), suggesting that the CD4^+^/CD8^+^ ratio may not always be an accurate biomarker or predictor of patient outcome when high stress (cortisol) levels are factored into the equation.

### 4.2 Neuroinflammatory Responses to Mammary Tumour Growth

Our next aim was to determine if mammary tumour growth causes neuroinflammation in the brain. The tissue concentrations of six well‐characterised cytokines were investigated using the ELISA technique. The myriad effects of cytokines are dependent on their concentration, as well as their site and duration of action, and so classifying them as either ‘anti‐’ or ‘pro‐inflammatory’ is often an oversimplification of their complex mechanisms of action (Levin et al. 2021; Stellwagen and Malenka 2006; Zipp et al. 2023). Interleukin‐2 (IL‐2) can act as a neurotrophin and supports the development of neurons and glia (Awatsuji et al. 1993; Sarder et al. 1993), enhancing neurite branching (Sarder et al. 1996), dendrite development and synaptogenesis (Shen et al. 2010). In addition, low dose IL‐2 supports the survival of regulatory T cells (Tregs) which control inflammation and autoimmunity (Klatzmann and Abbas 2015). IL‐4 has been shown to dampen neuroinflammation and promote the polarisation of microglia toward an ‘M2‐like’ anti‐inflammatory phenotype, thus protecting neurons from apoptotic signals (Wang et al. 2024a). Moreover, attenuated IL‐4 levels in the hippocampus exacerbate age‐related deficits in long‐term potentiation (LTP) (Maher et al. 2005). We found that the levels of IL‐2 and IL‐4 in sub‐cortical brain regions remained unchanged in all experimental groups of mice (Figure 5A,B). The same could be said for IL‐6 levels in the prefrontal cortex (PFC) (Figure 5F); a cytokine that can have both deleterious and protective actions in the CNS, depending on the context (Biber et al. 2008; Gadient and Otten 1997). Notably, restraint stress alone caused an elevation in IFNγ in the PFC (Figure 5D). IFN‐γ plays a key role in microglial priming, promoting their proliferation (microgliosis), and upregulating the production and release of nitric oxide. Therefore, IFN‐γ can cause enhanced phagocytosis of synapses and lead to impairment of synaptic transmission and cognitive functions (Kann et al. 2022). Interestingly, stressed tumour‐bearing mice (CRSTumor) did not show an upregulation of IFN‐γ suggesting that high levels of circulating corticosterone may be immunosuppressive and thus reduce neuroinflammation in the PFC. Indeed, the CRSTumor mice also displayed an attenuated rise of TNFα in the PFC (81.4 ± 8.8 pg/mg) compared to CANTumor mice (127.1 ± 6.6 pg/mg), although their TNFα levels were still elevated compared to Ctrl (37.7 ± 3.6 pg/mg), CRSRegress (48.6 ± 6.0 pg/mg) and CRSReject (41.1 ± 6.9 pg/mg) groups of mice (Figure 5E). Therefore, we can conclude that mice that displayed sustained tumour growth had elevated concentrations of TNFα in the PFC. There are a number of potential explanations for this finding. For example, tumour‐associated macrophages and other immune cells may release TNFα in response to tumour growth (Wang et al. 2024b) and this increase in systemic inflammation may be detectable by ELISA in brain tissue. Alternatively, mammary tumour growth may cause dysregulation of immune cells (e.g., T lymphocyte activation) which circulate and modulate the activity of endothelial cells and pericytes that wrap CNS neurovasculature (Li et al. 2022; Zhang et al. 2021). Thus, through indirect actions, immune cells may signal to resident glial cells in the PFC to increase TNFα release (Han et al. 2021). While neuroinflammatory signals may originate from the immune system, sustained tumour growth may also facilitate the release of cancer‐associated biochemical factors (or extracellular vesicles) from tumour cells into the bloodstream that then cross the BBB and act directly on neurons and glial cells in the PFC to induce TNFα release (Busatto et al. 2021). TNFα is a pleiotropic cytokine that has been demonstrated to enhance synaptic scaling and plasticity at low concentrations, but at higher levels, it has also been shown to inhibit long‐term potentiation of synaptic transmission and disrupt memory formation (Maggio and Vlachos 2018; Stellwagen and Malenka 2006). More experiments are required in order to elucidate the molecular mechanisms that cause raised TNFα levels in the PFC of CANTumor mice.

Finally, mice in which tumours did not develop (CANReject and CRSReject) displayed higher levels of the anti‐inflammatory cytokine, IL‐10, in the midbrain, thalamus and striatum compared to mice that developed tumours or those in which tumours regressed (Figure 5C). IL‐10 can attenuate pro‐inflammatory cytokine production by microglia, thus protecting astrocytes and neurons from inflammatory signals (Balasingam and Yong 1996; Ledeboer et al. 2002). IL‐10 also potentiates the production of transforming growth factor‐β (TGF‐β) by astrocytes which enhances microglial polarisation toward an anti‐inflammatory phenotype (Norden et al. 2014). IL‐10 acting on neurons via the IL‐10 receptor is associated with increased cellular survival (Zhou et al. 2009) and the regulation of adult neurogenesis (Pereira et al. 2015; Perez‐Asensio et al. 2013). Thus, IL‐10 is an important mediator of the complex crosstalk between microglia, astrocytes and neurons. We speculate that the CANReject and CRSReject groups of mice (i.e., 19% of those inoculated with Py230 cancer cells) may have mounted a robust immunogenic cell death (ICD) response that triggered a heightened ‘anti‐inflammatory’ physiological state which yielded increased levels of IL‐10 in the brain (and potentially other organ systems) with the function of protecting the host against any harm that could be caused by a systemic anti‐tumoural defence response (Fucikova et al. 2020; Showalter et al. 2017). Further studies are clearly required to elucidate the mechanisms leading to elevated IL‐10 levels in the brain of mice that have been inoculated with mammary tumour cells but have overcome that significant immune challenge.

### 4.3 Hippocampal Proteomic Changes in Response to Cancer

Given that both tumour growth and psychological stress had demonstrable effects on the immune system and the neuroinflammatory state of the mouse brain, our next goal was to conduct a proteomic screen of the hippocampus; a brain region that plays a key role in regulating both stress and memory formation (McEwen et al. 2016). We found 108 downregulated proteins in the hippocampus of CANTumor mice compared to Controls (Figure 6C). Interestingly, a cluster of 14 proteins involved in mitochondrial respiration, oxidative phosphorylation and electron transport chain‐coupled ATP synthesis were downregulated in CANTumor mice (Figure 6O). This suggests that mammary tumour growth may cause dysregulations in mitochondrial functioning in the hippocampus and disrupt the ability of neurons and glia to generate energy in the form of ATP (Choi and Han 2021). Downregulation of similar sets of proteins have been noted in the hippocampus of ageing mice compared to young controls (Navarro et al. 2008; Navarro and Boveris 2007). Mitochondrial dysregulations may be a common biomarker of senescence‐associated molecular changes in ageing CNS cells as well as those exposed to tumour‐derived biochemical factors (Luo et al. 2020; Miwa et al. 2022). We also found downregulations in a protein cluster made up of Slc25a4, Vdac1 and Vdac2 (Figure 6Q). Slc25a4 is an ADP:ATP antiporter that imports ADP into mitochondria and exports ATP to fuel cell functions (Hoshino et al. 2019). Together with the voltage‐dependent anion‐selective channel proteins, Vdac1 and Vdac2, which form channels between the mitochondrial outer membrane and the plasma membrane; this cluster of proteins may be involved in regulating lipid metabolism, necroptosis and cellular senescence in conjunction with the larger cluster of 14 mitochondrial respirasome proteins. Vdac1 has also been shown to regulate mitophagy and apoptosis (Ham et al. 2020). Bcl‐2 and Bcl‐xL molecules inhibit apoptosis by binding to Vdac1 and inhibiting the cytosolic transport of apoptotic signalling proteins, such as cytochrome *c* (Abu‐Hamad et al. 2008; Pastorino et al. 2002; Shimizu et al. 2000, 1999). In contrast, Bax and BID, which are proapoptotic molecules, bind to and open the Vdac1 pore and promote the release of cytochrome *c* into the cytosol (Baines et al. 2007; Shimizu et al. 2001, 2000). Vdac1 also binds inositol trisphosphate receptors (IP_3_R) at the endoplasmic reticulum, forming a mitochondria‐associated ER membrane (MAM) that facilitates calcium transport into the mitochondria following increases in ER calcium levels. Therefore, changes in these important regulatory proteins in the hippocampal neurons of CANTumor mice could indicate increased susceptibility to necroptosis (Figure 6Q).

In addition to mitochondrial dysregulations, CANTumor mice demonstrated decreases in synaptic plasticity‐related proteins in the hippocampus. This included a cluster of 6 proteins (Prkar1b, Prkacb, Ppp1r1b, Atp1b1, Gria3, Cpt1c) associated with AMPA and NMDA receptor activation and downstream signalling pathways regulated by PKA activity and cAMP‐mediated phosphorylation of the CREB1 transcription factor (Figure 6P). NMDA‐mediated upregulation of CREB transcriptional activity is arguably the most well characterised pathway driving long‐term synaptic plasticity events at the synapse and is essential for learning and long‐term memory formation in the hippocampus (Suzuki et al. 2011). This result provides a molecular framework that may shed some light on the pathophysiological mechanisms that underlie cancer‐related cognitive impairment. Indeed, we also found a decrease in Slc1a2 and Slc1a3 proteins (excitatory amino acid transporters [EAAT] 2 and 1, respectively) in CANTumor mice. These proteins are predominantly expressed by astrocytes and localise at the tripartite synapse to uptake excess L‐glutamate and L‐aspartate released by neurons during chemical synaptic transmission (Arriza et al. 1994). A decrease in EAAT1 and EAAT2 may increase the levels of glutamate overflow at the synapse and predispose neurons to excitotoxicity (Anderson and Swanson 2000; Diamond and Jahr 2000). Over time, however, reduced glutamate uptake will lead to a decrease in glutamine production by astrocytes and, therefore, a lack of the precursor molecule required to produce more glutamate within neurons (Andersen et al. 2021; Mahmoud et al. 2019). Therefore, chronic downregulation of EAAT transporters could exacerbate the low levels of AMPA and NMDA channel activity and decrease CREB1‐mediated LTP signalling events in neurons (Citri and Malenka 2008). Overall, these results could help to explain the brain fog and memory impairments often reported by people with various forms of non‐CNS cancers, such as triple‐negative breast cancer (Schagen et al. 2014).

### 4.4 Hippocampal Proteomic Changes in Stressed Tumour‐Bearing Mice

The next key aim of this study was to assess the impact of stress, in combination with breast cancer, on the hippocampal proteome. To investigate this, we first assessed hippocampal proteomic changes in stressed (RS) versus control (Ctrl) mice (Figure 7). Restraint stress led to the downregulation of 119 proteins and the upregulation of 32 proteins in the hippocampus. Interestingly, only 35 of these downregulated proteins were also lower in the CANTumor mice when compared to controls, suggesting that psychological stress impacts the hippocampal proteome in a distinct way to the physiological stress of tumour burden. The commonly downregulated proteins in RS and CANTumor mice included Prkar1b, Prkacb and GluR3 (Gria3) which are members of a cluster of proteins (Figure 7Q) involved in signalling events, such as PKA activation, downstream of AMPA and NMDA channel opening. As discussed above, this may lead to reduced CREB1 phosphorylation and disrupted long‐term synaptic plasticity in hippocampal neurons (Alberini 2009). We speculate that Prkar1b, Prkacb and GluR3, commonly downregulated in both restraint stress and in a model of TNBC, may be novel drug targets to enhance cognitive impairment caused by the stress and anxiety of a cancer diagnosis (Wong et al. 2014; Wu et al. 2007).

We also found that stress upregulates amyloid precursor protein (App) (Figure 7K) and downregulates transthyretin (Ttr) (Figure 7G), suggesting that chronic stress may lead to altered processing of amyloid peptides in the hippocampus. Ttr has been demonstrated to reduce Aβ‐induced toxicity in hippocampal neurons and in mouse models of Alzheimer's disease (Alemi et al. 2017; Silva et al. 2017). Interestingly, fibroblast growth factor 12 (FGF12) (Figure 7I) and seizure 6‐like protein 2 (Sez6l2) (Figure 7H) were completely ablated from the hippocampus of RS mice. Recent reports have shown that triple‐knockout of the Sez6 family of proteins (Sez6, Sez6L and Sez6L2) leads to a decrease in hippocampal dendritic spine density and a shift toward immature spine morphologies in the cortex of mice. Moreover, these KO mice demonstrated enhanced responses to stress, impaired working memory, and deficits in short‐term spatial memory (Nash et al. 2020). Reduced FGF12 levels have also been measured in the prefrontal cortex of female rats whose mothers had been subjected to a prenatal stress paradigm for three successive generations (McCreary et al. 2016). These rats demonstrated heightened anxiety in behavioral tasks, reduced hippocampal volume, and decreased neuronal cell densities in the hippocampus and cortex. Therefore, the downregulation of both FGF12 and Sez6l2 in the hippocampus of RS mice in the present study could help to explain how chronic stress often impairs learning and memory formation (Tomar and McHugh 2022).

Our main goal, however, was to investigate the impact of a psychological stressor (i.e., inescapable restraint) combined with the pathophysiological stress of mammary tumour growth on the hippocampal proteome. As such, we next looked at proteins that were up‐ or down‐regulated in the brains of CRSTumor mice compared to CANTumor mice (Figure 8). We found 178 proteins upregulated and 97 proteins downregulated in CRSTumor versus CANTumor mice. Gene ontology (GO) enrichment analysis suggested that proteins related to neurodegenerative processes, oxidative phosphorylation, and mitochondrial stress‐induced pathways were disrupted in stressed tumour‐bearing mice (Figure 8D). Notably, a large cluster (44) of functionally‐related proteins were upregulated in the hippocampus of CRSTumor mice (Figure 8J). One third (15) of these were ribosomal proteins which are linked to translational activity in cells (Kang et al. 2021; Wang et al. 2015). This process requires energy in the form of ATP and so this protein cluster also included 13 mitochondrial‐associated proteins known to be involved in the electron transport chain (Nolfi‐Donegan et al. 2020). Previous studies have shown that a range of chronic behavioural stress paradigms designed for rodents can lead to mitochondrial dysfunction in key cognitive brain regions, such as the prefrontal cortex and hippocampus, and that reactive oxygen species (ROS)‐mediated cellular stress can cause lipid peroxidation and neuronal apoptosis (Kaplan et al. 2023). Indeed, chronic stress in tumour‐bearing mice led to the upregulation of 3 proteins (Cpt1c, Faah, Abhd12) involved in lipid metabolism (Figure 8H). Studies have shown that disruption of Abhd12 causes elevations in lyso‐phosphatidylserine (lyso‐PS) lipids in human cells and the CNS of mice. Abhd12 is highly expressed in macrophages and microglia and plays a key role in regulating neuroinflammation (Ichu et al. 2020; Ogasawara et al. 2019). Loss‐of‐function mutations in Abhd12 cause the neurological disease PHARC (Polyneuropathy, Hearing loss, Ataxia, Retinitis pigmentosa and Cataract), and so the upregulation in Abhd12, noted here (Figure 8H), could potentially be a neuroprotective response to the activation of chronic stress‐induced signalling pathways in the hippocampus of CRSTumor versus CANTumor mice. Interestingly, we also found a significant downregulation in a cluster of 5 proteins potentially involved in synaptic plasticity (Ncam2, Prnp, Grb2, Crk and Crkl). The adapter proteins Crk and Crkl can relay signals from activated receptor tyrosine kinases (and non‐receptor tyrosine kinases) to various downstream effectors via protein–protein interactions with their SH2 and SH3 domains (Park and Curran 2008). The secreted extracellular protein, Reelin, is known to activate integrin signalling through a pathway involving the Crk protein family members and this promotes neuronal adhesion to extracellular matrix molecules, such as fibronectin (Sekine et al. 2012). Downregulation of these 5 proteins in the CRSTumor hippocampus may represent a pathological sign that neuronal processes which facilitate neurite extension and synaptic plasticity are dysregulated in chronically stressed cancer‐bearing mice.

Although not part of a protein cluster, we also noted that the G protein‐coupled receptor 37‐like 1 (Gpr37l1) featured in the top 25 most downregulated proteins in the hippocampus of CRSTumor versus CANTumor mice. Gpr37l1 is widely expressed in astrocytes and in a subpopulation of oligodendrocyte precursor cells in the mouse brain. Gpr37l1‐mediated signalling has been demonstrated to modulate astrocyte‐mediated regulation of extracellular glutamate concentration and reduce the sustained activation of neuronal NMDA receptor activity during ischaemic events (Jolly et al. 2018). Moreover, neuronal death increases by 40% in brain slices from Gpr37l1^−/−^ mice compared to wild‐types in an ex vivo model of ischaemia. Gpr37l1 is also involved in regulating neural plasticity by promoting adaptive myelination and the maturation of neural circuits in adulthood (An et al. 2021). Therefore, the 3.7‐fold downregulation of Gpr37l1 in the hippocampus of CRSTumor mice may represent a novel drug target for neuroprotection and improving cognitive function in stressed patients diagnosed with breast cancer.

### 4.5 Hippocampal Proteomic Changes in Response to Tumour Regression

A serendipitous feature of our dataset was the ability to compare proteomic changes in the hippocampus of mice in which tumours regressed versus mice in which tumours developed and continued to grow, or did not develop at all. A somewhat unexpected pattern emerged in the data which seems to suggest that the hippocampal proteomes of mice in which tumours initially developed and then spontaneously regressed (CANRegress and CRSRegress) are more similar to mice in which mammary tumours developed (CANTumor and CRSTumor) compared to the hippocampal proteomes of mice in which tumours never developed (CANReject and CRSReject). We found that there were 298 significantly upregulated proteins and 7 downregulated proteins in CANRegress versus CANReject mouse brain tissue (Figure 9C). Some of the upregulated proteins were plasticity‐related molecules, such as mGluR5 (Danjo et al. 2022), CamK2a (Cai et al. 2023), synaptophysin (Janz et al. 1999) and Snap25 (McKee et al. 2010; Selak et al. 2009) (Figure 9E–P). Indeed, we found a large cluster (57) of functionally‐related proteins composed of regulators of neurotransmission and vesicular exocytosis, as well as signal transduction molecules, Rho GTPase effectors, and ribosomal proteins involved in translation (Figure 10A). Taken together, the observation that these synaptic plasticity‐associated proteins are elevated in the hippocampus of CANRegress mice versus CANReject mice suggests that mice which rejected cancer cell inoculation may show greater deficits in cognitive function. While this suggestion is clearly speculative since we have no behavioural data to back up this claim, it is also not what we expected given that the CANReject mice showed higher levels of the anti‐inflammatory cytokine, IL‐10, in subcortical brain structures (Figure 5C). There is evidence to suggest that IL‐10 promotes synaptic plasticity (Chen et al. 2016; Nenov et al. 2019). However, as mentioned previously, the effects of cytokines in the brain are highly dependent on their concentration, site of action and duration of action (Mocellin et al. 2003; Yirmiya and Goshen 2011). A recent study has highlighted that chronic IL‐10 overproduction by astrocytes reduces hippocampal neurogenesis and impacts spatial memory formation in mice (Sanchez‐Molina et al. 2022). Therefore, although CANReject mice mounted a successful immune challenge that killed Py230 cells injected into the mammary fat pad, this heightened immunogenic cell death (ICD) response may lead to negative effects on synaptic transmission in key cognitive areas of the brain, such as the hippocampus (Bellingacci et al. 2023). Interestingly, myelin‐related proteins including Plp1, Mog, Mbp and Cnp were downregulated in CANReject mice compared to CANRegress mice, suggesting that hippocampal myelination may also be dysregulated in mice in which tumours did not develop (Figure 10F).

We next compared the hippocampal proteomes of CANTumor mice versus CANRegress mice to assess if the spontaneous regression of mammary tumours after 7–9 weeks of initial growth led to demonstrable differences in the brain compared to mice which fully developed mammary tumours (Figure 11). While there were clearly significant differences between the hippocampal proteomes of CANRegress and CANTumor mice, these groups were more similar to one another than to the CANReject group of mice. Somewhat surprisingly, they were also more similar to control mice than to the CANReject group (Figure 11A,B). We found that there were 60 proteins downregulated and 3 proteins upregulated in the hippocampus of CANTumor versus CANRegress mice (Figure 11C). Again, several of the downregulated proteins have clear connections to the regulation of synaptic plasticity (Figure 11E–P). A cluster of 9 functionally related proteins involved in mitochondrial processes, ATP synthesis, and oxidative phosphorylation were also downregulated in the hippocampus of CANTumor mice (Figure 11S). Taken together, this could mean that although the mammary tumours of CANRegress mice spontaneously receded after an initial period of tumour growth, there may be long lasting changes to the hippocampal proteome that could, in turn, impact cognition, mood or behaviour in negative ways.

Finally, to investigate this hypothesis further, we compared the hippocampal proteomes of CRSTumor versus CRSRegress mice (Figure 12). We found that there were 17 down‐ and 4 up‐regulated proteins in the hippocampus of CRSTumor versus CRSRegress mice, thus highlighting the similarity in protein expression between these two groups (Figure 12C). While the hippocampal proteomes of CRSTumor and CRSRegress mice were remarkably similar, there were still some key proteins of interest downregulated in chronically stressed mice whose tumours continued to grow larger over the 12‐week timeframe (Figure 12F–J). These included calretinin (Calb2) (Gulyás et al. 1996), neuromodulin (GAP43) (Holahan et al. 2010), Tppp3 (Huang et al. 2017), Tmsb10 (Li et al. 2023), and cadherin‐13. The cell adhesion molecule, cadherin‐13, is predominantly localised to presynaptic terminals of GABA‐ergic inhibitory interneurons in the hippocampus, and its dysregulation is a known risk factor for neurodevelopmental and psychiatric disorders, such as ADHD, autism and depression (Kiser et al. 2019; Rivero et al. 2015). Cdh13^−/−^ mice display increased inhibitory tone and dampened levels of synaptic transmission in CA1 pyramidal neurons. As such, Cdh13^−/−^ mice display an imbalance in the ratio of excitatory to inhibitory neurotransmission in the hippocampus and deficits in learning and memory formation (Rivero et al. 2015). Calretinin, on the other hand, functions to buffer intracellular Ca^2+^ levels in dentate granule cells and thus plays a key role in synaptic plasticity and calcium‐dependent synaptic signalling events. Calb2^−/−^ mice display hyperexcitability in the dentate gyrus and a reduced K^+^ current due to downregulated surface expression of Kv4.1 subunits. This leads to impaired pattern separation in the dentate gyrus and thus impacts learning and memory formation (Kim et al. 2021). Taken together, our data show that although the hippocampal proteomes of CRSTumor and CRSRegress mice are relatively similar, there are a few key differences which suggest that a chronic psychological stressor on top of the physiological stress of mammary tumour growth may cause further detrimental effects to cognition, learning and memory formation.

### 4.6 A Comparison With Other Mouse Models of Non‐CNS Cancers

**FIGURE 13:**
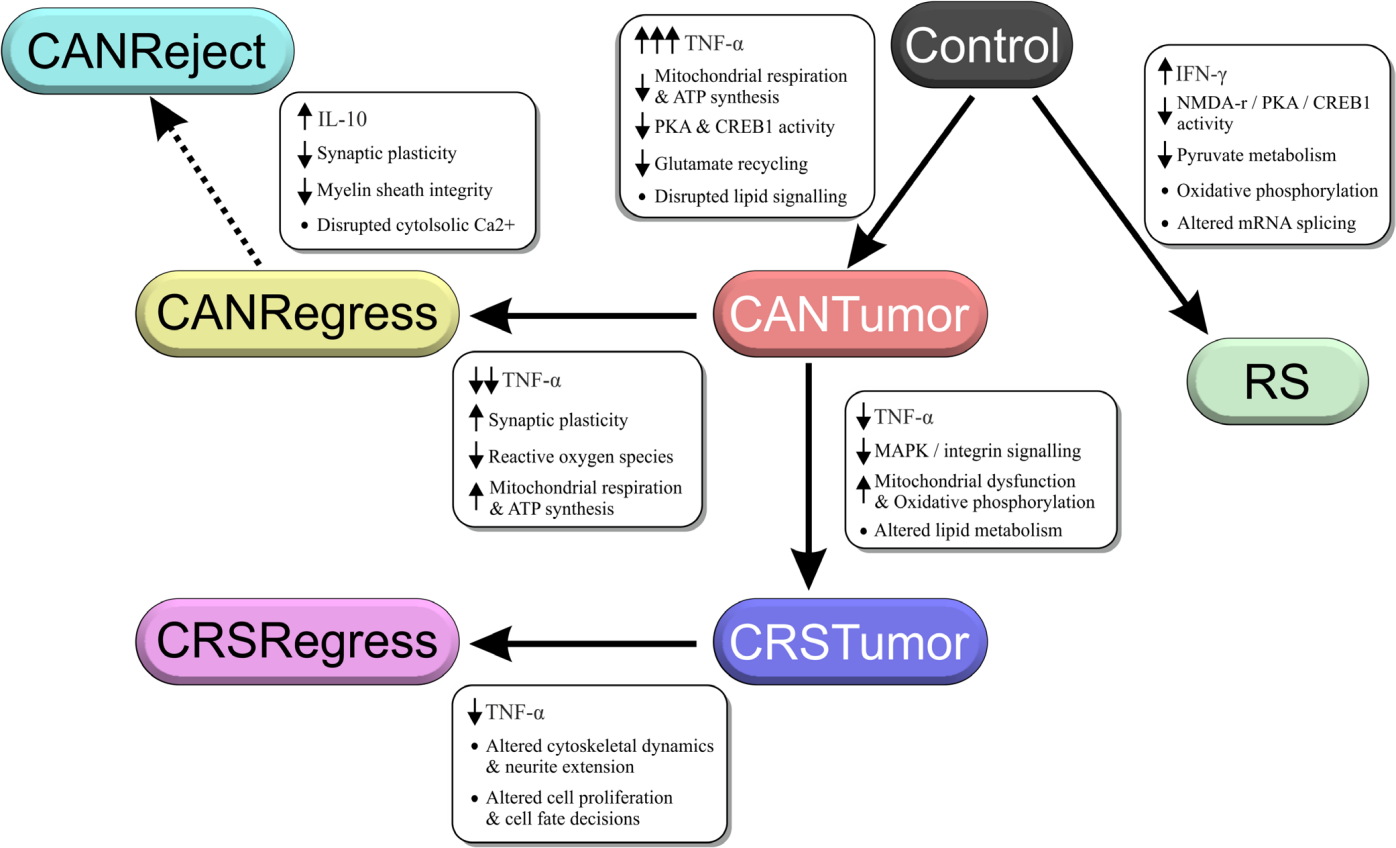
Schematic diagram summarising the impact of restraint stress, cancer, and tumour regression on the hippocampal proteome. The arrow directions indicate the changes that occur in the hippocampal proteome in the destination group of mice compared to the origin group. For example, Control ➔ RS describes changes in the restraint stress group compared to control mice. The white box beside each large arrow lists the main proteins and pathways impacted. Small north‐facing arrows = up‐regulation. Small south‐facing arrows = downregulation. RS (restraint stress); CANTumor (mice that developed tumours); CRSTumor (mice that developed tumours and experienced restraint stress); CANRegress (mice that developed tumours which spontaneously regressed); CRSRegress (mice that developed tumours which spontaneously regressed and also experienced restraint stress); CANReject (mice that were inoculated with cancer cells but did not develop tumours); IL‐10 (interleukin‐10); Ca2+ (calcium); TNFα (tumour necrosis factor‐α); ATP (adenosine triphosphate); PKA (protein kinase A); CREB1 (cyclic adenosine monophosphate responsive element binding protein 1); IFN‐γ (interferon‐γ); NMDA‐*r* (N‐methyl‐D‐aspartic acid receptor); MAPK (mitogen‐activated protein kinase).

The main findings of this study are summarised in a simplified schematic diagram (Figure 13). Investigations into the effects of non‐CNS cancers on the brain, before anti‐neoplastic treatments have begun, are sparse. However, this is a relatively new field of research which has been gaining traction in the last few years. A recent study has shown that mammary tumours (i.e., 4T1.2 adenocarcinoma cells) in BALB/c mice cause activation of microglial cells in the hippocampal dentate gyrus, the arcuate nucleus of the hypothalamus, and the amygdala (McCaffrey et al. 2022). Interestingly, astrocytic GFAP expression also decreased in the prefrontal cortex of these BALB/c mice, suggesting an altered neuroinflammatory state caused by tumour burden. Strehle et al. (2024) also investigated neuroinflammation in an orthotopic syngeneic mouse model of breast cancer (i.e., mammary 67NR non‐metastatic tumour cell line in BALB/c mice). They found that tumour growth increased microglial Iba and astrocytic GFAP expression in the amygdala and hippocampal brain regions. This apparent neuroinflammatory state was accompanied by increased ICAM‐1, IL‐6 and IL‐1β in the amygdala and increased IL‐1β expression in the hippocampus, as measured by qPCR (Strehle et al. 2024). Using a syngeneic cell line (E0771) in female C57Bl/6 mice, Netherby‐Winslow et al. (2023) showed that serum IL‐1β and IL‐2 were elevated 4 weeks post‐tumour cell inoculation, and this correlated with a decrease in hippocampal neurogenesis (Netherby‐Winslow et al. 2023). This finding may be explained by an earlier study by Yan et al. (2022) who demonstrated that 4T1 triple‐negative breast cancer cells transplanted into BALB/c mice led to reduced levels of serotonin, norepinephrine, and BDNF in the hippocampus. Moreover, NF‐κB activity‐induced expression of the pro‐inflammatory cytokines IL‐1β, IL‐6 and TNFα were also elevated in the hippocampus of these tumour‐bearing mice (Yan et al. 2022). Taken together, there is an increasing body of evidence to suggest that non‐CNS cancers can lead to a state of neuroinflammation in the brain which may impact cognitive functions and mood.

## 5 Conclusion

Although breast cancer has been the most studied type of tumour in the context of pre‐treatment CRCI, the literature on this condition is rapidly growing in people that develop other types of cancer (e.g., small cell lung cancer, gastrointestinal cancers, acute myelogenous leukaemia, Hodgkin lymphoma, pancreatic cancer and testicular cancer) (Baekelandt et al. 2016; Meyers et al. 2005; Simó et al. 2015; Trachtenberg et al. 2018; Vardy et al. 2015; Wefel et al. 2011). We present here a starting point for future studies aimed at elucidating the cellular and molecular mechanisms of cancer‐related cognitive impairment. It will be important to complement this study with experiments that investigate the impact of different types of cancers on the brain, such as primary tumours of the ovaries, prostate, liver, or blood cancers, for example. Are there common CRCI‐inducing mechanisms between the different types of cancers, or are the proteomic changes we report here specific to mammary tumours grown in young female mice? Moreover, it will be important, going forward, to investigate sex and age differences in how the brain responds to tumour growth and whether this data will translate to the human condition and prove to be clinically‐relevant for identifying novel drug targets to treat *brain fog* or memory impairments in cancer survivors. Many questions remain to be answered in this nascent, but flourishing, field of cancer neuroscience.

## Author Contributions

**Myrthe Mampay:** investigation, writing – original draft, methodology, validation, visualization, writing – review and editing, formal analysis, data curation, software. **Gheed Al‐Hity:** investigation, methodology, data curation. **Sara O. Rolle:** validation, visualization, formal analysis. **Walla Alzboon:** formal analysis, software. **Nicolas A. Stewart:** methodology, writing – review and editing, supervision, investigation, validation, software, data curation. **Melanie S. Flint:** conceptualization, funding acquisition, methodology, writing – review and editing, project administration, resources, supervision, investigation. **Graham K. Sheridan:** conceptualization, funding acquisition, writing – original draft, methodology, visualization, writing – review and editing, project administration, resources, supervision, data curation, software, formal analysis, investigation, validation.

## Ethics Statement

All experiments were conducted in compliance with the ARRIVE guidelines.

## Consent

Informed consent was achieved for all subjects, and the experiments were approved by the local ethics committee.

## Conflicts of Interest

The authors declare no conflicts of interest.

## Supporting information


Data S1.

## Data Availability

All data that support the findings of this study are included in the results section and figures of the main article and in the supplemental data file. Raw data are available from the corresponding author upon reasonable request.
